# Apparent Velocity Extraction from Quadrupole Acoustic Logging While Drilling: CNN-Based Moveout Regression

**DOI:** 10.3390/s26216977

**Published:** 2026-11-04

**Authors:** Cong Xu, Chuang Hei, Mingzhang Luo

**Affiliations:** 1 School of Electronic Information and Electrical Engineering, Yangtze University, Jingzhou 434023, China; 2022730060@yangtzeu.edu.cn (C.X.); heichuang@yangtzeu.edu.cn (C.H.); 2 National Demonstration Center for Experimental Electrotechnics and Electronics Education, Yangtze University, Jingzhou 434023, China

**Keywords:** acoustic logging while drilling (LWD), quadrupole, convolutional neural network, moveout regression, apparent velocity, slowness–time coherence, threshold mask

## Abstract

**Highlights:**

• A median relative velocity error of 2.51% ± 0.16% is obtained by five-fold cross-validation on kinematic synthetic gathers; an error of 2.11% with *R*^2^ = 0.947 is obtained on the held-out synthetic test.
• On the same held-out gathers, the lower median error (1.57%) is obtained with STC, whereas the gross-error rate (relative error > 20%) is reduced from 3.88% to 0.50% via the CNN.
• Processing is performed 3.4× faster by the 266 KB model than by STC processing on the same 16-core CPU (≈21× when the CNN is run on a desktop GPU, a ratio that is not hardware-normalized), indicating potential suitability for edge implementation.

**Abstract:**

In slow formations, dispersive quadrupole guided waves are used in acoustic logging while drilling (LWD), and a global coherence maximum that can switch between competing arrivals is picked by conventional slowness–time coherence (STC) processing. In this study, we train a lightweight convolutional neural network (CNN) on kinematic synthetic gathers, regress the inter-receiver moveout directly from quadrupole array gathers, and convert the estimated moveout to an apparent quadrupole-mode velocity. The conversion uses an empirically set effective spacing of 0.0725 m (nominal spacing of 0.100 m), so independent calibration is required for the absolute velocity scale. Five-fold cross-validation yields a median relative velocity error of 2.51% ± 0.16%, and the held-out test yields 2.11%, with *R*^2^ = 0.947. On the same held-out gathers, a lower median error (1.57%) is attained by STC, whereas fewer gross errors (relative error > 20%; 0.50% versus 3.88%) are produced by the CNN; sharp degradation of the CNN is shown by stress tests when secondary events are stronger (*A*_s_/*A*_t_ ≥ 0.75) or the SNR is lower (≤5 dB) than that in training. On a measured slow-formation interval, numeric and image-inversion median apparent velocities differ by 13.9% before and 0.05% after 2.5–4.5 kHz band-pass filtering (pointwise *r* = 0.36). A control trained on the measured STC log reaches r = 0.53 on held-out records, but only r = 0.20 under depth-blocked cross-validation. On the same 16-core CPU, the 266 KB model is 3.4× faster than STC (≈21× with the CNN on a desktop GPU, which is not hardware-normalized). The results support kinematic moveout regression with potential edge suitability; absolute formation shear-wave velocity is not independently validated.

## 1. Introduction

Acoustic logging while drilling (LWD) provides formation elastic-wave velocities in real time, supporting geosteering, pore-pressure prediction, and geomechanical evaluation without a dedicated wireline run [1,2,3]. A central difficulty of while-drilling measurement and downhole intelligent monitoring is that both the telemetry bandwidth and the downhole computing power are limited in the while-drilling scenario, so processing is best performed locally and in a lightweight manner near the sensor; this practical constraint is the starting point of the method design in this paper [4].

In slow formations, where the formation shear-wave velocity is lower than the borehole fluid’s acoustic velocity, the refracted shear head wave no longer exists, and monopole measurements therefore lose the direct route to the formation shear wave. In this case, multipole excitation must be used to excite a dispersive guided wave in the borehole, whose phase velocity at low frequencies approaches the formation shear-wave velocity, from which the shear slowness can be inverted [5,6,7]. For an axially equispaced receiver array, the formation shear information reduces to the constant time shift with which this guided-wave packet advances across the traces, i.e., the time difference between adjacent receivers.

For four decades, the standard method for reading this time shift from array waveforms has been slowness–time coherence (STC) [8,9], i.e., scanning over a slowness–time grid and picking the coherence maximum, with the coherence measure inherited from semblance in multichannel seismic analysis [10]. This paradigm faces two prominent limitations in field measurements. First, the hard decision to take the global maximum: once a secondary coherent arrival captures the peak, the velocity log along depth exhibits isolated outliers. Second, the cost of the grid search grows with the slowness–time resolution; it is small on desktop hardware (0.6–2.9 ms per gather in this study, against a 4 s record interval) but matters for high-throughput reprocessing and for power- and compute-limited downhole processors, where telemetry bandwidth is limited, and lightweight inference near the sensor is preferred [11,12,13]. Classical improvements have proceeded along two routes, i.e., statistical efficiency and dispersion bias. In the former, statistical efficiency and resolution are improved through parametric spectral estimation and maximum-likelihood frameworks [14], whereas in the latter, systematic bias is removed through dispersion correction, such as dispersive STC processing [15] and high-resolution dispersion inversion [16]. Neither, however, departs from model fitting followed by extremum picking. Hence, hard-decision outliers and the computational burden persist, leaving room to replace this paradigm with a learned continuous mapping.

Deep learning has been increasingly applied to acoustic and geophysical interpretation using logging curves, array waveforms, and signal-derived images [17,18]. In the approaches most closely related to the present work, labeled training data are generated by synthetic forward modeling, and the learned relationship is transferred to measured observations [19,20], or data domains are bridged by domain adaptation [21]. In a related application, deep learning has been used to regress formation slowness directly from LWD acoustic wave modes [22], and physics-informed networks have been developed for dispersive dipole acoustic processing [23,24]. Other, less direct applications include sonic-transit-time reconstruction from conventional logging curves [25,26], waveform-based first-arrival picking [27,28,29], and regression of physical parameters from time–frequency or signal images [30,31,32]. These studies target formation slowness, dispersion characteristics, or arrival picks; they give less attention to regressing a purely kinematic quantity—the inter-receiver moveout from quadrupole LWD array gathers under kinematic synthetic supervision.

The present method follows a synthetic-supervision strategy but targets only the kinematic structure of the quadrupole guided-wave packet. The CNN is trained on kinematic synthetic gathers with prescribed inter-receiver moveout and is applied to measured gathers without measured velocity labels. The estimated moveout is converted to an apparent quadrupole-mode velocity using an empirically set effective receiver spacing. Unlike the approaches of [16,23,24], this method neither models nor inverts dispersion, so it is a label-free kinematic alternative to scan-and-pick processing rather than a substitute for dispersion-aware inversion. As a control, the same architecture is also trained from scratch on the measured commercial STC velocity log, allowing the influence of STC-derived, potentially noisy labels [33,34,35] to be examined.

Relative to the work of [22,23,24], the increment of this study is therefore specific and modest: kinematic synthetic moveout supervision, direct regression from quadrupole array gathers, label-free application to measured LWD data, and a lightweight single-pass network are combined, and this combination is evaluated against classical moveout estimators on identical synthetic gathers and on one measured quadrupole dataset from a slow-formation experimental well. Because no independent velocity measurement is available for that interval, we treat the measured results as apparent-velocity extraction and consistency checks rather than as validation of absolute formation velocity, and we do not claim numerical superiority over published methods evaluated on different data. Table 1 summarizes the relationship between the proposed method and representative acoustic shear-wave processing methods.

The main contributions are as follows:

1. Inter-receiver moveout is regressed from quadrupole array gathers by a lightweight CNN trained only on kinematic synthetic gathers, with median relative velocity errors of 2.51% ± 0.16% in five-fold cross-validation and 2.11% on the held-out test. On the same gathers, its median error is higher than that of the STC (1.57%), but its gross-error rate is lower (0.50% versus 3.88%).
2. The failure boundaries of the CNN outside its training distribution are delineated by controlled stress tests on secondary-event amplitude and signal-to-noise ratio.
3. The STC log is fitted by a measured-STC-supervised control under random splitting (*r* = 0.53) but not under depth-blocked validation (pooled *r* = 0.20; mean per-fold *r* = 0.006), showing that the apparent learnability of STC labels stems from near-neighbor leakage; because the STC log is derived from the same measurements, independent validation of the synthetically supervised method is not provided by this control.
4. Single-pass inference is performed 3.4× faster by the 266 KB model than by the STC on the same 16-core CPU (≈21× when the CNN is run on a desktop GPU), indicating potential for edge implementation; embedded deployment was not evaluated.

## 2. Methods and Principles

A synthetically supervised, lightweight CNN regresses inter-receiver moveout directly from array gathers, then converts the estimated moveout to an apparent quadrupole-mode velocity using the array geometry. The kinematic structure of the quadrupole measurement is exploited in this design: in slow formations, the shear-wave information is carried by the quadrupole guided-wave mode, whose low-frequency phase velocity approaches the formation shear-wave velocity [5,6], and the observation of this mode on an axially uniform receiver array reduces to a single kinematic quantity—the constant time shift of the wave packet across traces, i.e., the inter-receiver moveout *a*.

Under the linear arrival-time model, the arrival time is a linear function of the receiver index, and its slope is *a*. The apparent quadrupole-mode velocity *V*_app_ is obtained from the CNN-estimated moveout $\hat{a}$ and the effective receiver spacing:

(1) $$V_{\mathrm{app}}=\frac{\Delta z_{\mathrm{eff}}}{|\hat{a}|\;\Delta t}$$

where $\left|\hat{a}\right|$ is the absolute value of the CNN-estimated inter-receiver moveout (samples per channel), Δ*t* is the sampling interval, and Δ*z*_eff_ is the effective receiver spacing used for velocity conversion. The physical receiver spacing is Δ*z*_nom_ = 0.100 m, whereas Δ*z*_eff_ = 0.0725 m is an empirically set value. Because *V*_app_ is proportional to Δ*z*_eff_, the absolute scale of the field output is fixed by this value and is therefore, not independently calibrated.

**Figure 1:**
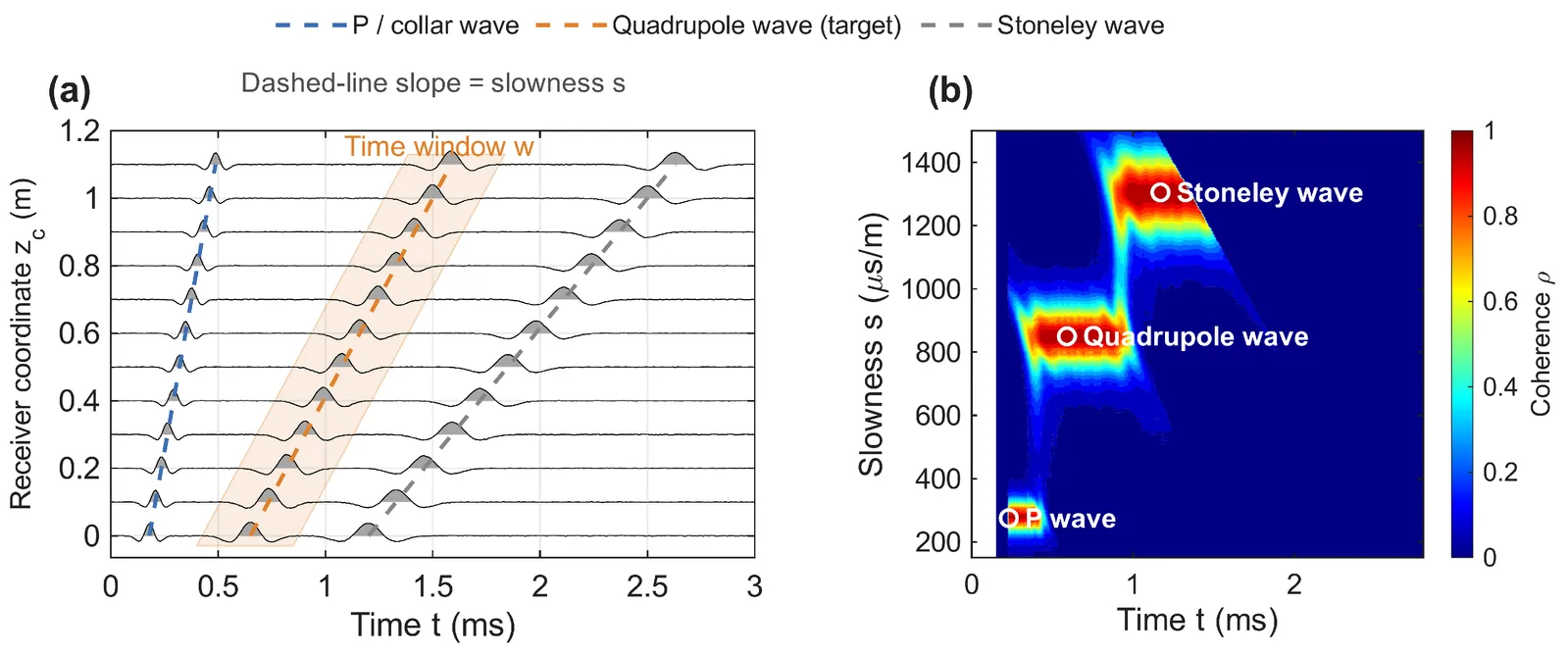
Principle of STC processing: (**a**) 12-trace array waveforms; (**b**) slowness–time coherence map.

In standard STC processing, a grid search is performed over the slowness–time plane, and the global coherence maximum is taken [8,9]; this hard decision can switch wholesale to a secondary arrival in the wavefield, appearing as outlier spikes on the velocity curve along depth. This processing is illustrated in Figure 1 with a sample quadrupole array record: (a) the 12-trace array waveforms are shown, in which the borehole mud/collar wave, the target quadrupole shear arrival, and the Stoneley wave arrive in succession; the target wave packet of interest advances linearly across the trace index with a constant time shift, the slope of the dashed line corresponds to the slowness, and its time window is marked by the shaded band; (b) the coherence distribution over the slowness–time plane is shown, where a coherence peak is formed by each of several arrivals, and the slowness at the global maximum is the STC estimate. The STC output is thus taken from the global maximum of a multi-peak coherence surface. Semblance-type coherence measures are near-optimal under the premise of a single coherent arrival, whereas coexisting secondary coherent arrivals such as reflections from adjacent wells, collar waves, and coda are often present in measured LWD wavefields, so the coherence surface is multi-peaked; when a secondary peak approaches the target peak, the hard decision can switch wholesale between peaks. This mechanism motivates replacing the scan-and-pick step with continuous regression.

**Figure 2:**
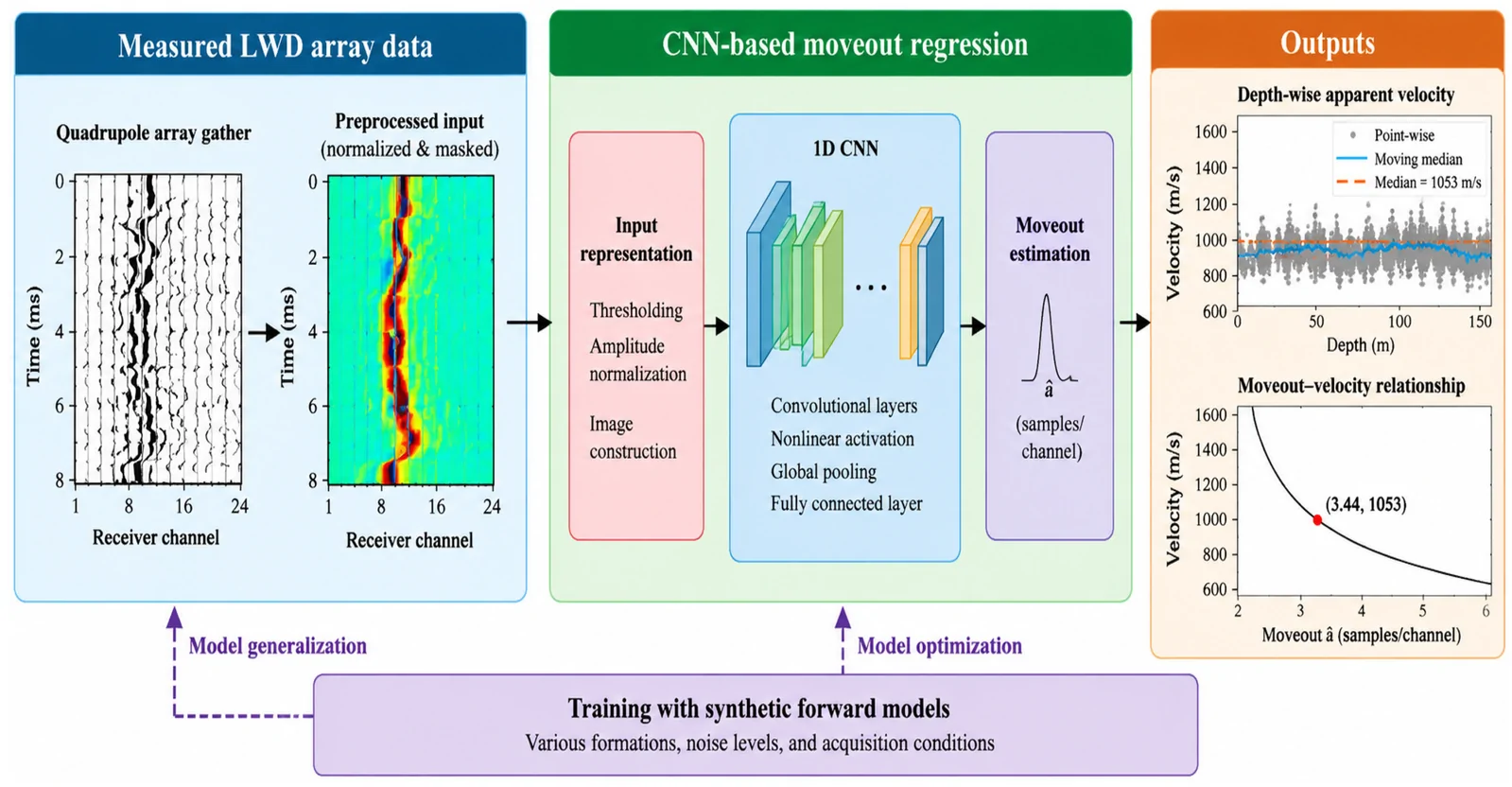
Processing pipeline from array gathers to apparent-velocity extraction.

Accordingly, the network is trained on kinematic synthetic gathers with known injected inter-receiver moveouts [19,20,21] and regresses moveout from threshold-masked gathers; training uses no per-record labels nor reference velocity curves, but fixes the output scale to an empirically adopted reference value. Figure 2 presents the processing workflow. We first organize the measured waveforms into a 12 × 400 array gather, then apply threshold-mask gating to emphasize the coherent wave packet and suppress low-amplitude background components. The lightweight CNN then maps the masked gather to the inter-receiver moveout, and Equation (1) converts the estimated moveout to the apparent quadrupole-mode velocity. The synthetic gathers are kinematic: moveout, wavelets, secondary events, and noise are prescribed, but dispersion, collar waves, attenuation, and borehole geometry are not modeled.

### 2.1. Theoretical Foundations

#### 2.1.1. Quadrupole Measurement

In slow formations, the formation shear-wave velocity is lower than the borehole fluid’s acoustic velocity; the critical refraction condition is no longer satisfied at the borehole wall, so the refracted shear head wave gliding along the wall does not exist, and monopole direct arrivals cannot provide formation shear-wave information directly. Multipole excitation must then be employed, of which the quadrupole is the most typical: rather than a body-wave head wave, a dispersive guided-wave mode propagating along the borehole axis is excited by a quadrupole source in a fluid-filled borehole; its phase velocity varies with frequency and approaches the formation shear-wave velocity at low frequencies, so the formation shear slowness can be inverted from the low-frequency phase velocity of this mode [5,6]. The multipole-source theory of borehole multipole acoustic measurements is described in [7] and provides a systematic treatment of borehole acoustic guided waves [36].

Formally, the formation quadrupole mode corresponds to the *n* = 1, *n* = 2 branch of the period equation for a fluid-filled borehole. The dispersion relation of this branch is denoted as

(2) *D*_2_(*k*,*ω*; *V*_s_,*V*_p_,*ρ*,*V*_f_,*ρ*_f_,*r*_w_) = 0,  *v*_Q_(*f*) = *ω*/*k*

where *k* is the axial wavenumber, and *ω* is the angular frequency; *V*_s_, *V*_p_, and *ρ* are the formation shear-wave velocity, compressional-wave velocity, and density; *V*_f_ and *ρ*_f_ are the acoustic velocity and density of the borehole fluid; and *r*_w_ is the borehole radius. For a given frequency, the phase velocity *v*_Q_(*f*) of this mode is determined by the root satisfying Equation (2) [5,7,36]. The branch has a cutoff frequency *f*_c_ and propagates only for *f* > *f*_c_; as $f\to f_{c}^{+}$, the phase velocity approaches the formation shear-wave velocity *V*_s_ from below. It falls progressively lower as frequency increases [5,6,36].

The quadrupole mode is inherently dispersive, and its phase velocity approaches the formation shear-wave velocity only at low frequencies. A single-moveout conversion that neglects dispersion can therefore underestimate the shear-wave velocity [15,16]. Dispersion is not modeled by the present method: frequency-dependent phase velocity is included in neither the synthetic gathers nor the CNN, and the empirically set effective spacing cannot be read as a dispersion correction: because the quadrupole-mode velocity lies below the formation shear-wave velocity, the converted velocity would be raised by a dispersion correction, whereas it is lowered by 27.5% by Δ*z*_eff_ = 0.0725 m relative to the nominal spacing. At the nominal spacing, all variants already exceed the median of the measured STC log (processed with the same dispersive mode) by 32–51%; thus, the origin of this scale factor is unexplained. The output is therefore an apparent quadrupole-mode velocity rather than a dispersion-corrected formation shear-wave velocity.

The steel drill collar can also act as an acoustic waveguide and introduce collar-related quadrupole interference. The low-frequency operating band is intended to lie below the collar quadrupole-mode cutoff, suppressing collar propagation while retaining a measurable formation quadrupole response [5,6]; in practice, residual collar arrivals may remain (Figure 1a), and they were not modeled explicitly in this study.

#### 2.1.2. Linear Arrival-Time Model

The measured guided-wave mode is assumed to advance along the borehole axis with slowness *s*, and *z_c_* is used to denote the axial coordinate of the *c*-th receiver *c* = 1, …, *N*); the arrival time on that receiver is then given by

(3) *τ_c_* = *τ*_0_ + *s* (*z_c_* − *z*_1_)

The arrival time is a linear function of trace index: the intercept *τ*_0_ is determined by the excitation time and the propagation path from the source to the first receiver, whereas the slope *s* is determined by the apparent slowness of the measured guided-wave mode, which approaches the formation shear slowness at the low-frequency end.

#### 2.1.3. Slowness–Time Coherence

Semblance, a coherence measure from multichannel seismic analysis [10], is used in STC processing [8,9]. It is constructed as follows: on the slowness–time plane spanned by the candidate slowness and the window start time, the traces are moveout-corrected, aligned, and stacked according to the time shift implied by each candidate slowness, and the coherence is then computed within a fixed time window as the ratio of the energy of the aligned stack to *N* times the sum of the individual trace energies, where *N* is the number of receivers. The waveform of the *c*-th receiver is denoted by *x_c_* and its axial coordinate by *z_c_* (*c* = 1,…N); for candidate slowness *s* and window start time *t*_0_, the coherence is given by

(4) $$\rho (s,t_{0})=\frac{\sum_{t\in w}{\left(\sum_{c=1}^{N}x_{c}(t+s\;z_{c})\right)}^{2}}{N\sum_{t\in w}\sum_{c=1}^{N}x_{c}^{2}(t+s\;z_{c})}$$

where window *w* starts at *t*_0_, and a fixed number of samples is included; by the factor of *N* in the denominator, *ρ* ∈ [0, 1] is ensured, and the measure is made invariant to an amplitude scale common to all traces. The candidate slowness *s* and the inter-receiver moveout are in one-to-one correspondence through *a* = *s* Δ*z*/Δ*t*.

Slowness, or equivalently, inter-receiver moveout, is estimated by STC by locating the global maximum of the semblance surface over the slowness–time plane. Although semblance is invariant to common amplitude scaling, receiver-dependent gain differences can reduce constructive alignment; per-trace amplitude normalization is a common remedy, but we did not apply it here, instead normalizing each gather as a whole. For a single nondispersive arrival contaminated by additive white Gaussian noise, and when the in-window energy is approximately independent of candidate slowness, maximizing semblance approaches maximum-likelihood slowness estimation, providing the statistical basis for conventional STC [8]. Parametric and maximum-likelihood approaches can further improve slowness resolution and statistical efficiency [14].

This favorable behavior becomes less reliable in measured LWD wavefields containing multiple coherent arrivals, including collar/tool waves, formation coda, and secondary reflections. These components can generate competing coherence peaks, making global-maximum selection sensitive to the locally dominant event. When peak amplitudes are similar, the selected maximum may switch, and small waveform perturbations can abruptly change the estimated moveout. Multi-peak extraction, slowness tracking, and quality-control procedures can mitigate this behavior, but they do not completely remove ambiguity under strong event competition. Quadrupole-mode dispersion further complicates estimation because the coherence peak can shift, and variations in spectral content can bias apparent slowness [15,16]. In addition, computational cost increases with slowness–time search resolution. Although important limitations of conventional STC are addressed by statistical-efficiency [14] and dispersion-aware [15,16] methods, explicit search or model-fitting procedures are generally retained.

#### 2.1.4. Moveout Estimation

The precision with which the inter-receiver moveout can be read is statistically limited; independent of the specific estimator; and jointly determined by the waveform energy, bandwidth, and array geometry. For the model of a single arrival of known waveform in additive white Gaussian noise (one-sided power spectral density *N*_0_), the Cramér–Rao lower bound for an unbiased moveout estimate $\hat{\tau }$ is

(5) $$\mathrm{var}(\hat{\tau })\;\geq \;{\left[(2E/N_{0})\;\beta^{2}\right]}^{-1}$$

where *E* is the signal energy, and *β* is the root-mean-square angular bandwidth of the signal (the square root of the second moment of angular frequency weighted by the signal energy spectrum) [37]; the corresponding bound for the passive two-channel stochastic-signal case is given in [38]. Unbiased moveout estimators are constrained by this bound; when the waveform is unknown, the bound can only be larger, so Equation (5) remains a valid lower bound, whereas bias is traded for variance in a biased regression mapping, which is not directly constrained by it. Such limits serve as benchmarks in parametric spectral-estimation processing aimed at statistical efficiency [14], connecting to the semblance near-optimality argument in the preceding subsection. It is shown by Equation (5) that attainable moveout precision tightens as the signal-to-noise ratio and effective bandwidth increase: the quadrupole wave packet is band-limited, so the readout precision of a single-pair inter-receiver moveout is limited, but not impossible in principle.

The moveout-slope estimate is further tightened by array redundancy. Under the linear arrival-time model, if the arrival-time readings of *N* equally spaced receivers are each affected by independent errors of variance $\sigma_{\tau }^{2}$, then for a least-squares fit of the straight line of arrival time versus trace index, the variance of the slope (slowness) estimate scales as [39]

(6) $$\frac{12\;\sigma_{\tau }^{2}}{N\;(N^{2}-1)\;\Delta z^{2}}$$

At a given spacing, the variance decreases approximately with the cube of the number of receivers; at a given number of receivers, it tightens with the square of the array aperture.

### 2.2. Data and Extraction Method

#### 2.2.1. Measured Dataset

The measured data were acquired with a quadrupole-array acoustic logging-while-drilling (LWD) tool in the physical experimental well of the National Key Laboratory of Deep Oil and Gas, China University of Petroleum (East China) [40]. The receiver section is a 12-receiver axial array; Table 2 summarizes the acquisition and processing constants. The investigated interval is a slow formation, where the formation shear-wave velocity is lower than the borehole-fluid velocity, so no refracted shear head wave exists, and quadrupole excitation is required.

Three record sets of the same interval are used. (i) All 2198 records of the numeric quadrupole file (26,376 traces from 12 receivers, 1024 samples per trace) are included in the numeric domain. We removed the mean from each trace over the full record; retained the first 400 samples, which contain the complete target wave packet; and normalized each gather by its maximum absolute amplitude. This is the primary input. (ii) The 2000 available archived waveform plots (one per record) are included in the image-inversion domain. The embedded color bar was sampled to build a color-to-amplitude lookup table spanning +1 to −1, the waveform region was cropped and divided into a 12 × 400 grid, and the median RGB value of each cell was converted to amplitude by nearest-neighbor matching, with near-gray background cells set to zero; no DC removal was applied. Because this domain is a lossy re-encoding of the same measurements, only consistency checks are supported. (iii) A total of 1032 records are covered by the commercial STC log, in which one velocity is provided per record. No depth field is stored in either file; records were acquired at 4 s intervals (one 8 s gap after record 252). The sets are therefore paired by record index: image record i corresponds to numeric record i + 1 (i = 1, …, 2000), the one-record offset at which inter-domain waveform correlation is maximized, and the STC log corresponds to image records 1–1032. This pairing is statistical, and the records’ identities cannot be proven. Successive records alternate between two acquisition states (lag-1 waveform correlation ≈ 0.03, lag-2 ≈ 0.5–0.7), and this period-2 alternation also appears in the velocity output. All constants in Table 2 were applied over the whole interval without depth-dependent adjustment.

#### 2.2.2. Lightweight CNN

The estimator takes the threshold-masked 12 × 400 gather as input and regresses the inter-receiver moveout directly. After normalizing to a maximum absolute amplitude of 1, it retains samples above the threshold and suppresses lower-amplitude components. The lightweight CNN includes four convolutional blocks, global average pooling, a 64-unit fully connected ReLU layer, dropout (*p* = 0.2), and a linear output, with 68,065 learnable parameters. Anisotropic convolution and pooling preserve the cross-trace phase structure along the 12-channel axis while progressively compressing the temporal dimension.

**Figure 3:**
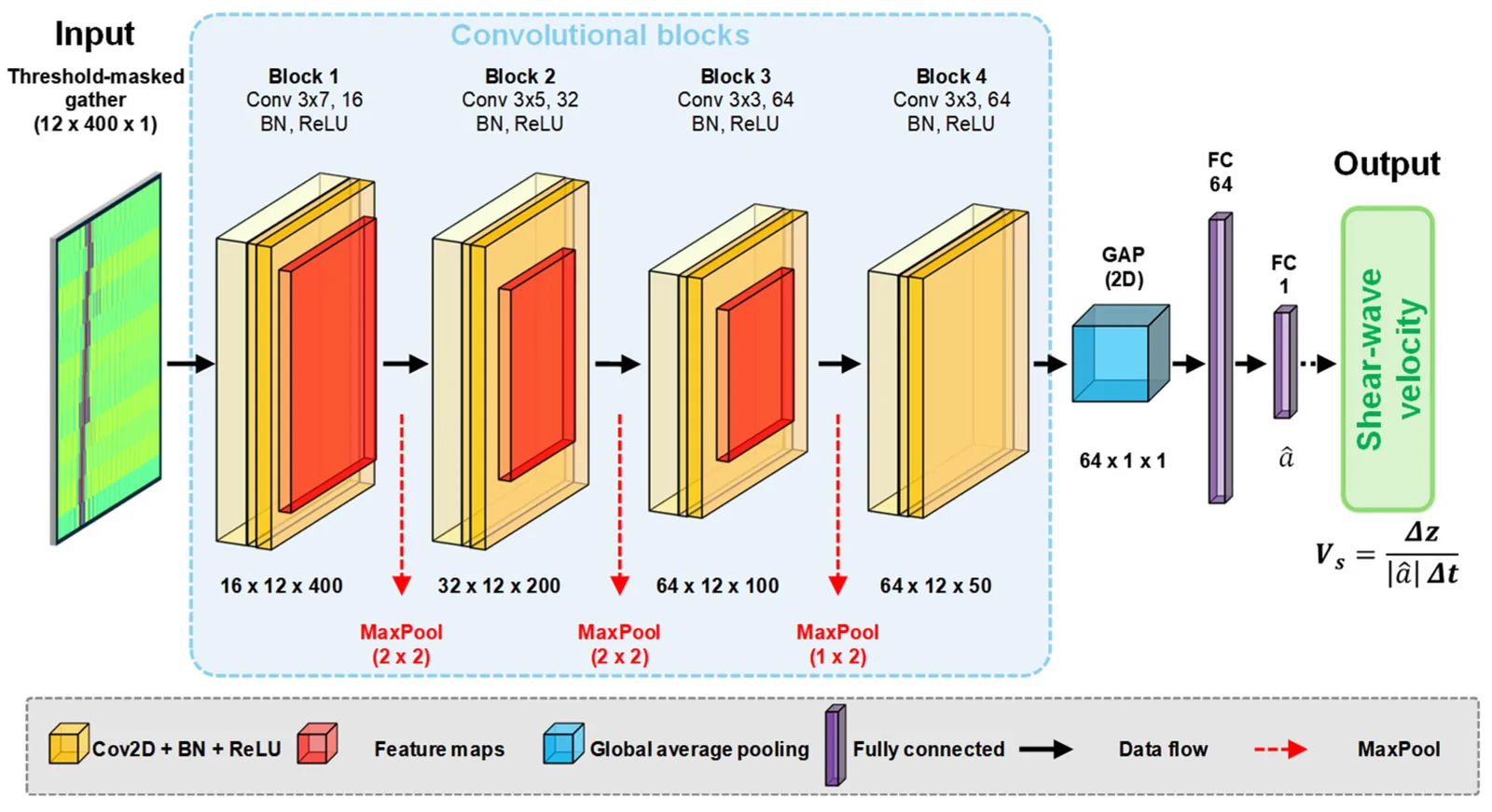
Architecture of the regression CNN.

Figure 3 shows the architecture: the threshold-masked 12 × 400 gather passes through anisotropic convolutional blocks that extract the wave packet’s 2D morphology along the channel and time axes; after global average pooling, the fully connected layers output a scalar inter-receiver moveout. No search over a slowness grid is involved; the forward pass is the estimate.

The layers are listed in Table 3. The network has 68,065 learnable parameters (saved model file of 266 KB), far fewer than the approximately 0.49 M parameters of the Attention U-Net segmentation front-end used in the processing comparison, because only a single kinematic scalar has to be regressed after wave-packet gating by the threshold mask without trainable weights. Inference consists of a single deterministic forward pass, making the model a potential candidate for near-sensor edge computing; embedded deployment was not evaluated. For numeric-domain records, trace-wise offsets are suppressed by per-trace mean removal, but receiver gain differences are not compensated for; no DC removal is applied to image-inversion records nor synthetic gathers. Each 12 × 400 gather is then normalized by its maximum absolute amplitude, retaining relative amplitudes among the 12 channels, and thresholded at *θ* = 0.5.

A deterministic function *f_w_* is implemented by the network and determined by its learnable parameters *w*, which are distinct from the mask threshold *θ*; the threshold-masked gather *X* is mapped to the moveout-magnitude estimate $\hat{a}=f_{w}(X)$. Given *M* paired samples {(*X_i_*,*a_i_*)}, the parameters are fitted with the mean squared error (MSE) in the moveout domain as the objective:

(7) $$w_{*}=\arg\min_{w}\frac{1}{M}\sum_{i=1}^{M}{\left(f_{w}\left(X_{i}\right)-a_{i}\right)}^{2}$$

Under MSE training, an MSE-optimal regression mapping over the training distribution is approximated by the network; in the population limit, this mapping is the conditional expectation $f_{w_{*}}(X)\approx E[a|X]$ of the moveout, given the gather *X*. This mapping is only approximately realized by a finite network trained on finite kinematic synthetic data and only within the training distribution. The intended inductive bias is that the moveout *a* appears as a constant phase slope of the wave packet along the channel axis, which is extracted layer by layer by the anisotropic kernels and aggregated into a single scalar by global average pooling.

The kinematic invariances are also encoded explicitly into the estimator by this design. Through per-gather normalization and the threshold mask, *f_w_* is made insensitive to the overall amplitude scale, which is of the same origin as the scale invariance of semblance; after aggregation by global average pooling, the output is rendered approximately invariant to a global time shift of the wave packet by the translation equivariance of convolution [41], precisely corresponding to the fact that *a* does not depend on the absolute arrival time *τ*_0_. The estimate is therefore driven mainly by the relative cross-trace phase structure.

The essential difference from STC lies in the decision mechanism. The STC estimate is taken from the global maximum of a multi-peaked coherence surface so that when two peaks compete, the estimate can switch wholesale between them. In the present method, a continuous mapping composed of convolutions, ReLUs, and pooling aggregates evidence from the entire masked input into one estimate without a global argmax over a slowness–time grid (its max-pooling layers select only local maxima within small neighborhoods of the feature maps). This removes an important source of abrupt outliers, although large errors can still occur under severe interference, low SNR, or distribution shift. This mapping is fitted on synthetic data in the main line and on measured STC labels in the comparison variant.

#### 2.2.3. Training on Kinematic Synthetic Gathers

The main-line CNN was trained only on kinematic synthetic gathers with known inter-receiver moveout, so no measured velocity labels are needed [19,20,21]. A target event built from a Gabor wavelet with prescribed moveout and randomized onset, one or two coherent secondary events, an optional coda, and band-limited Gaussian noise is included in each 12 × 400 gather (Table 4). Target velocities drawn from U(760, 1520) m/s are converted to moveout with Δ*z*_eff_ = 0.0725 m, giving |*a*| = 2.38–4.77 samples/channel. Relative velocity errors on synthetic data do not depend on the value of Δ*z* because the truth and the estimate both scale as 1/|*a*|; only errors expressed in m/s scale with Δ*z*.

The velocities of secondary events are drawn from the same range, and their onsets are placed 25–135 samples after the earliest target arrival on channel 1, so the target may be preceded or overlapped by them on some channels (overlap within 45 samples on at least one channel in about 55% of events). All parameters are sampled independently for each gather; through this domain randomization, the network is encouraged to learn the cross-trace propagation pattern rather than specific amplitudes or arrival positions [42]. The generator is kinematic rather than a full-waveform borehole model: frequency-dependent dispersion, collar wave, attenuation, tool eccentricity, borehole geometry, formation elastic parameters, and modal interference are not included, and adaptation to measured data is not performed.

The training, validation, and test sets are composed of 4000, 800, and 800 gathers generated with seeds 1–4000, 20,001–20,800, and 40,001–40,800, respectively (gather i generated with rng(seed)); rng was used for the training run. The CNN in Table 3 was trained from scratch with Adam on the MSE of |a| in samples/channel (unnormalized) for 40 epochs, with a batch size 64, an initial learning rate of 0.001 halved every 12 epochs, and L2 regularization of 0.001; no early stopping was used, and the network with the lowest validation loss was retained. Weights were initialized with the MATLAB defaults (Glorot-uniform weights, zero biases; batch-normalization scale 1 and offset 0). Training was performed using MATLAB R2026a on an NVIDIA RTX 5080 GPU. For five-fold cross-validation, a separate pool of 5000 gathers (seeds 50,001–55,000) was randomly partitioned into five folds (rng 42); in fold k, 10% of the training portion was held out for validation (rng 100 + k), and 35 epochs were used for training with otherwise identical settings.

#### 2.2.4. Application to Measured Data

The trained network was applied to the measured records after preprocessing; one deterministic forward pass is required for each gather, and the estimated moveout is converted with Equation (1) into the apparent quadrupole-mode velocity *V*_app_. An effective spacing Δ*z*_eff_ = 0.0725 m is used for the conversion instead of the nominal Δ*z*_nom_ = 0.100 m, and this value was set empirically. With 0.100 m, the numeric-domain median was 1654 m/s (median moveout of 3.02 samples/channel); this median is rescaled by the factor *k* = 1200/1654 = 0.725 to a slow-formation reference value of 1200 m/s. Tang et al. (2015) [43] specify this formation shear-wave velocity in Table 1 of their cased-hole acoustic model. This value also matches the approximate reference provided by the experimental personnel for wells No. 5 and No. 9. It serves as a literature-supported empirical reference for velocity scaling, not as an independent velocity measurement for the investigated interval. We did not derive the effective spacing theoretically nor optimize it on an independent calibration interval; we fixed it after examining the measured STC log, although the adopted 1200 m/s differs from the STC median of 1095 m/s. The numeric-domain median of 1200 m/s is therefore reproduced by construction; we cannot corroborate the absolute scale by comparison with the measured STC log, and the outputs should be read as relative profiles requiring absolute-level calibration. Because *V*_app_ ∝ Δ*z*, all velocities are raised by 1.38% for an increase of 1 mm in Δ*z*. For Δ*z* = 0.0725, 0.080, 0.090, and 0.100 m, the median *V*_app_ is 1200, 1323, 1489, and 1654 m/s in the numeric domain; 1053, 1162, 1307, and 1452 m/s in the image-inversion domain; and 1168, 1288, 1449, and 1610 m/s in both domains after the 2.5–4.5 kHz band-pass; at 0.100 m, the measured-STC median is exceeded by 32–51% by all variants.

We obtained the measured numeric- and image-domain results and the U-Net variants from an earlier training run of the same architecture using an earlier generator version; its median error on the Table 5 test set is 2.63% (*r* = 0.956, 0.50% gross errors). For the band-passed variants, we use a separate network of the same architecture, retrained on synthetic gathers band-passed with the same 2.5–4.5 kHz filter. Numeric-domain medians of 1129–1225 m/s are obtained with five independently trained networks with the same Δ*z* (synthetic median errors of 2.2–2.6%), so the absolute level also depends, by around ±4%, on the training realization.

### 2.3. Comparison Protocols

#### 2.3.1. Measured-STC Supervision

For comparison, the measured STC log is used as the label for a supervised variant [33,34], following well-logging studies in which acoustic-log prediction is supervised by labels generated by deterministic relations [34]. On the 1032 image-inversion records covered by the STC log, a CNN with the main-line architecture is trained from scratch with the STC velocity *V*_STC_ as the target, using a random 826/206 split (rng 42); the 206 held-out records were also used for model selection and are therefore, not a fully untouched test set. *V*_STC_ is converted to the regression target by

(8) *a*=Δ*z*/(*V*_STC_ Δ*t*)

Δ*z* appears consistently in both the label encoding and the velocity decoding, so the converted velocity is independent of the specific value; velocities on the scale of the measured STC labels are output by the network.

In the population limit, the MSE-optimal regression function for Equation (7) is the conditional expectation, which decomposes as

(9) E[*V*_STC_∣*X*] = E[V^*^∣*X*] + E[*ε*∣*X*]

where V^*^ is the latent ground truth, and *ε* is the STC label error. This mapping is only approximated by a finite network trained on 826 records. Label spikes caused by secondary arrivals that capture the coherence peak are a minority, whose morphology–velocity relation conflicts with the majority; when they are not systematically associated with repeatable input morphology, the model cannot predict them from the input and averages them into the conditional mean rather than reproducing them point by point. However, because large residuals are weighted quadratically by MSE, the fitted mapping is still shifted by these label spikes in proportion to their frequency and magnitude. This is a statistical tendency, not a guarantee of robustness or physical accuracy, particularly outside the training distribution.

The expected variance reduction follows from the law of total variance [39]:

(10) Var(*V*_STC_) = E[Var(*V*_STC_∣*X*)] + Var(E[*V*_STC_∣*X*])

where the ideal regression function f_*_(*X*) = E[*V*_STC_∣*X*] is constrained by Var(f_*_(*X*)) ≤ Var(*V*_STC_), the difference being the conditional label variance that cannot be explained by the input; the weaker the explanatory power of the input, the stronger the shrinkage. A fitted network exhibits this shrinkage only approximately.

#### 2.3.2. Evaluation Protocol

Synthetic accuracy is reported on the 800 held-out test gathers as MAE, median relative velocity error with percentile bootstrap 95% confidence intervals (1000 resamples, seed 2026), fractions within 5% and 10%, r, and gross-error rate, where a gross error is a relative velocity error exceeding 20%; split stability is assessed by five-fold cross-validation. Measured profiles are summarized by the median and interquartile range (IQR) of raw point-wise values; the 21-record moving median is for display only.

The classical estimators (Table 6) used raw, unmasked test gathers with the same Δ*z*, with parameters fixed before evaluation and not tuned on the test set, except that GCC-PHAT was band-limited after failing over the full band (35.3% median error). The full 400-sample traces of the 11 adjacent receiver pairs, lags within ±10 samples, three-point parabolic refinement, and the median pair delay are used in cross-correlation (CC). GCC-PHAT [38] uses the same pairs and refinement, a 1024-point FFT, weighting R/(|R| + 10^−6^·max|R|), and zero weight outside 1.5–4.5 kHz; this baseline is favored by the generator-informed band. In the robust fit, each trace’s Hilbert-envelope maximum is picked within 25 samples after the first sample exceeding half the envelope maximum (parabolic refinement). The Theil–Sen slope (median of 66 slopes) is fitted. In STC [8,9], semblance is used in a 45-sample window over 700–1600 m/s in 25 or 5 m/s steps, with both moveout polarities, integer-sample shifts, and the global maximum over all window starts (no arrival-time gating).

In the stress tests, the 800 test seeds are regenerated with *A*_s_/*A*_t_ fixed between 0 and 1.0 or SNR fixed between 22 and −5 dB (training ranges of 0.20–0.55 and 10–22 dB), and the CNN (retrained with the same protocol) is compared with 25 m/s STC (Table 7). For threshold sensitivity, a network was retrained for each *θ* = 0.3, 0.4, …, 0.7 and applied with the same *θ*. In the cross-domain consistency analysis (Table 8), numeric and image-inversion outputs are compared before and after the 2.5–4.5 kHz band-pass under the index pairing, with bootstrap confidence intervals (5000 i.i.d. resamples for medians, in which the serial correlation of the records is ignored, and optimistic intervals are therefore obtained; moving blocks of 50 records for r). Attention U-Net versus threshold masking (the U-Net being trained on threshold-rule labels), the quadrupole combination (S1 − S2 + S3 − S4)/4, and a self-computed STC on band-passed numeric records are compared by their medians. All of these come from the same measurements, and we test only consistency.

For the measured-STC-supervised variant (Table 9), the metrics are the ratio of medians, median relative error, fractions within 5% and 10%, and r against a constant training-label-median baseline. In depth-blocked five-fold cross-validation, contiguous blocks of records are held out in acquisition order (no depth field is stored, so the record index is used as a proxy for depth); both the pooled out-of-fold r and the mean per-fold r (comparable with the classical regressors) are reported. Distributions are compared using bootstrap confidence intervals and the Brown–Forsythe test.

Runtime (Table 10) on the 1032 image-domain gathers is the mean ± SD of 10 repetitions after a warm-up run. The CNN was timed on the GPU and on the CPU, including host–device transfer but not masking; 16 CPU workers, a 45-sample window, and 25 or 5 m/s steps over 1000–3500 m/s at 0.100 m (≡725–2538 m/s at 0.0725 m) were used for STC. GPU–CPU ratios are not hardware-normalized; embedded hardware, power, activation memory, and end-to-end preprocessing were not evaluated.

## 3. Results

### 3.1. Inter-Receiver Moveout Regression Accuracy

**Figure 4:**
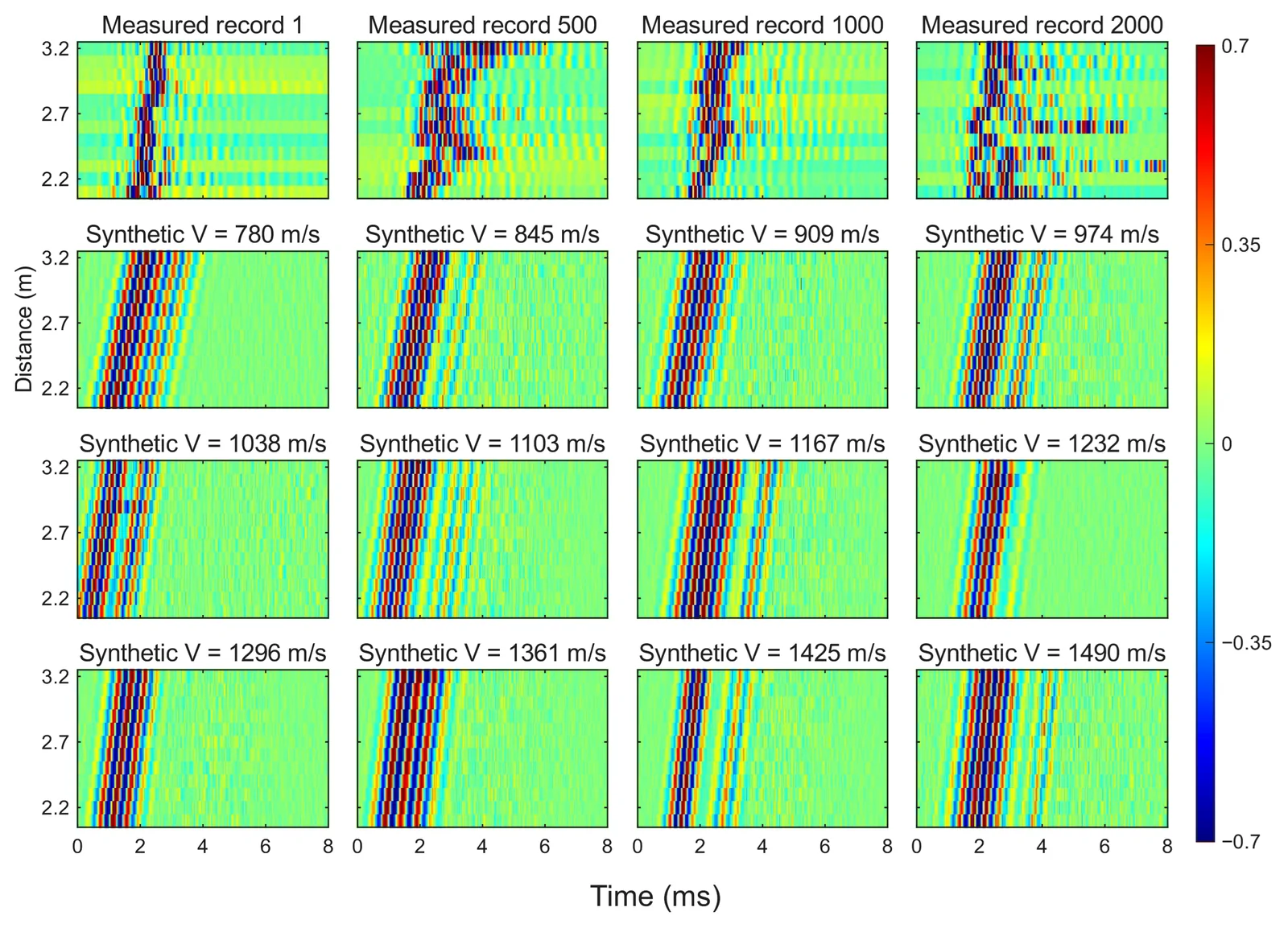
Synthetic training gathers versus measured gathers.

On the synthetic benchmark with known ground truth, the inter-receiver moveout is regressed by the network with a median relative velocity error of about 2–2.5% and negligible bias. For transfer to measured data, the kinematic synthetic gathers must be sufficiently similar to the measured gathers in the features the network relies on. Figure 4 compares representative measured quadrupole gathers with synthetic samples spanning apparent velocities from 780 to 1490 m/s. The measured examples show coherent wave packets with clear cross-receiver moveout, along with greater waveform irregularity and background interference, particularly in records 0500 and 2000. In the synthetic cases, increasing velocity reduces the cross-trace time shift, while the oscillatory wave-packet structure and duration remain qualitatively comparable to those of the measured gathers. The measured gathers nevertheless show more complex local amplitude and waveform variations not reproduced by the kinematic generator.

**Figure 5:**
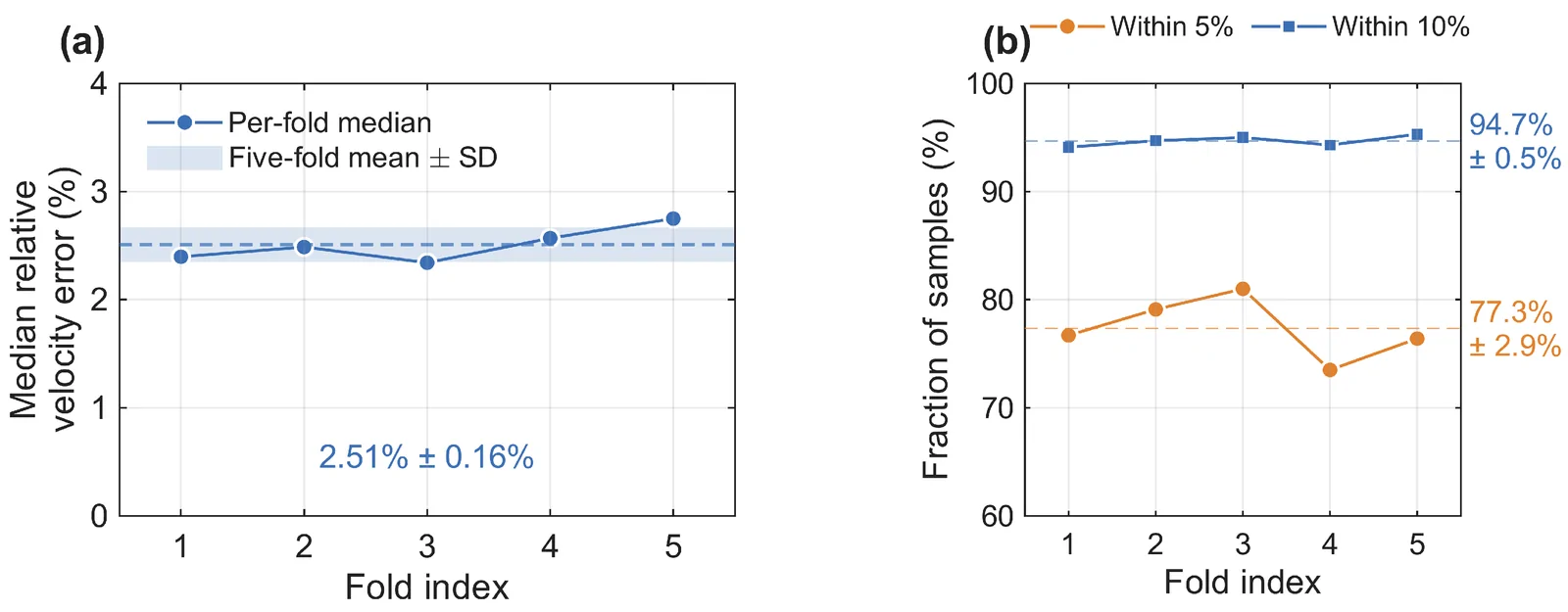
Five-fold cross-validation on synthetic gathers. (**a**) Per-fold median relative velocity error. (**b**) Per-fold fractions with relative error ≤ 5% and ≤10%.

The five-fold cross-validation of the synthetically supervised regression is presented in Figure 5, i.e., (a) the median relative velocity error of each fold, which fluctuates around the five-fold mean of 2.51% ± 0.16%; (b) the fractions within 5% and 10%, at 77.3% ± 2.9% and 94.7% ± 0.5%, respectively. The small cross-fold dispersion indicates insensitivity of accuracy to the data split. Cross-validation quantifies stability, while the held-out test set provides the point estimate.

**Figure 6:**
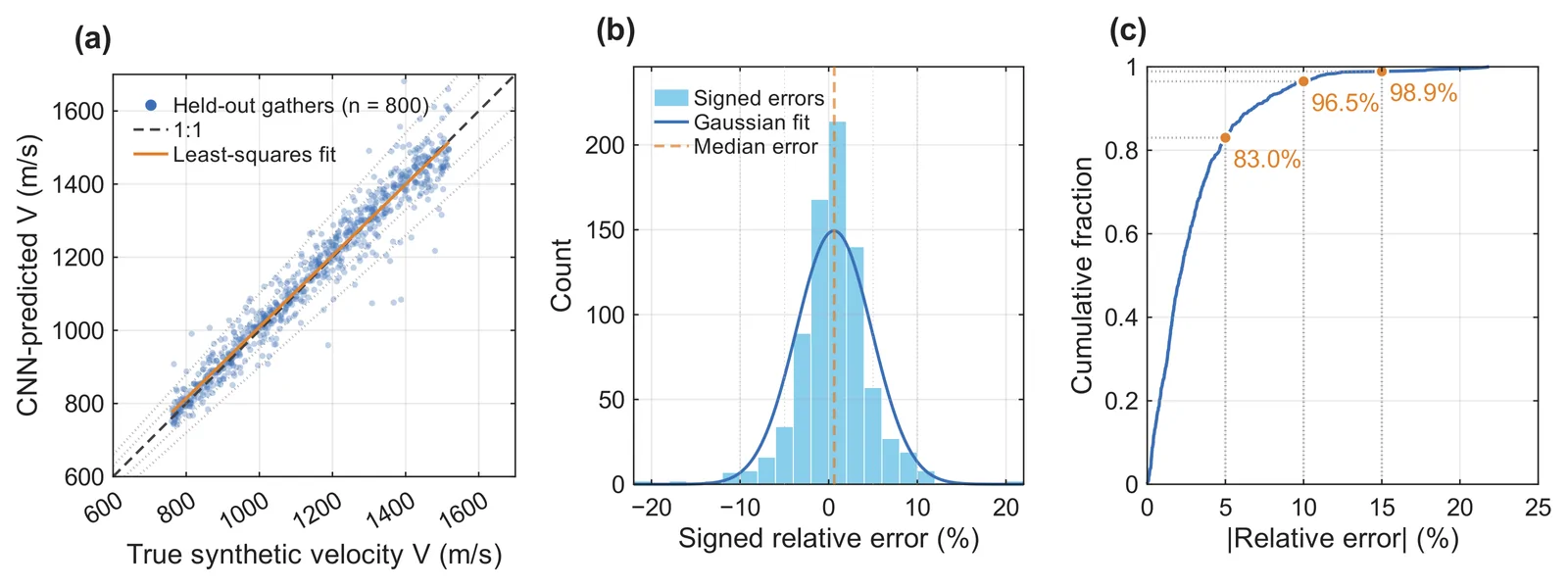
Inter-receiver moveout regression CNN on the synthetic test set. (**a**) Predicted versus true velocity. (**b**) Relative-error histogram with normal fit. (**c**) Cumulative relative-error distribution.

The held-out test set is evaluated in Figure 6: (a) predicted versus true velocity, with samples clustered along the 1:1 line and most of them inside the ±5%/±10% bands; (b) the signed relative error, centered near zero (median bias +0.63%) but heavier-tailed than a normal distribution (MAPE 3.02% versus a median of 2.11%; P95 of 9.0%); (c) the cumulative distribution of the relative error, with 83.0%, 96.5%, and 98.9% of samples within 5%, 10%, and 15%, respectively, and a P95 of 9.0%. In this synthetic distribution, the errors are therefore mainly small random fluctuations around the true values, with no systematic over- or underestimation.

The held-out metrics are summarized in Table 5: *R*^2^ = 0.947, *r* = 0.974, CCC = 0.973, RMSE = 51 m/s, MAE = 35 m/s, median relative velocity error = 2.11%, and gross-error rate = 0.50%. The held-out median error (2.11%) is lower than every five-fold cross-validation fold (2.34–2.75%; mean ± SD 2.51% ± 0.16%), whose networks were trained on 3600 gathers for 35 epochs. The network behind Table 5 and Table 6 was not archived; an error of 2.18% is obtained by an identical-protocol retrain on the same test set. Relative velocity errors do not depend on the receiver spacing used for conversion, whereas errors in m/s scale with it. The network applied to the unfiltered measured data is an earlier training run of the same architecture with an earlier version of the generator, and a separate band-pass-specific network is used for the band-passed variants; on the same test set, a median error of 2.63% (*r* = 0.956; gross-error rate 0.50%) is obtained with the earlier network.

In Table 6, the CNN is compared with conventional moveout estimators on the same 800 held-out gathers: cross-correlation, generalized cross-correlation with phase transform (GCC-PHAT) [38], robust moveout fitting, and STC [8,9]. Identical results were obtained with a wider STC grid (725–2538 m/s) or a single moveout polarity.

The lowest median relative error is obtained with STC (1.57% at 25 m/s and 1.30% at 5 m/s, versus 2.11% for the CNN), whereas the lowest MAE (34.8 m/s), RMSE (51 versus 91 m/s for 25 m/s STC), and gross-error rate (0.50% versus 3.88%), and the highest correlation (*r* = 0.974), are obtained with the CNN. A paired bootstrap at 25 m/s STC confirms both differences: the CNN median error is 0.54 percentage points higher [95% CI 0.33, 0.77], and its gross-error rate is 3.38 points lower [−4.88, −2.00]. Refining the STC grid from 25 to 5 m/s lowers the median error but not the gross-error rate, so the STC gross errors reflect wrong-peak selections rather than grid quantization. GCC-PHAT failed over the full band (median error 35.3%) because phase-transform whitening weights noise-only frequency bins equally; the band-limited version reported here uses knowledge of the generator band, so this baseline is favored. On these kinematic synthetic gathers, a slightly larger random error is thus traded for markedly fewer gross errors by the CNN. This comparison favors the CNN because the test gathers are drawn from the same generator as its training data, including the statistics of the secondary events, and because neither arrival-time gating nor slowness tracking is used by the STC baseline, the gross-error advantage therefore applies to ungated STC within this generator’s distribution.

Robustness was tested on gathers generated from the same 800 held-out seeds with either the secondary-to-target amplitude ratio *A*_s_/*A*_t_ (training range of 0.20–0.55) or the SNR (training range of 10–22 dB) fixed at each level. The CNN in these tests is an identical-protocol retrain (median error of 2.18% on the unmodified test set), and the 25 m/s grid is used for STC. As shown in Table 7, STC achieves a lower median error at every level, whereas the CNN produces fewer gross errors within the training distribution and up to *A*_s_/*A*_t_ = 0.75 (8.5% versus 15.88%). Outside the training distribution, the CNN degrades sharply: at *A*_s_/*A*_t_ = 1.00, its median error is 10.15%, and its gross-error rate is 20.88% (STC: 4.66% and 23.5%); at SNR = 0 and −5 dB, its median error rises to 7.43% and 12.13%, whereas STC stays below 2.1%, with gross-error rates of at most 0.13%. An independent earlier run with different seeds (400 gathers per level, *A*_s_/*A*_t_ from 0.55 to 1.0) agrees in direction. The CNN therefore suppresses gross errors from the wrong peak, but its random error is slightly larger, and it provides no guarantees outside its training distribution. In contrast, STC is markedly more robust to additive noise.

The mask threshold was examined by retraining and applying the network with *θ* = 0.3, 0.4, 0.5, 0.6, and 0.7. The synthetic median error is nearly flat from 0.3 to 0.6 (2.08–2.44%; 86.1–80.4%, within 5%) and degrades at 0.7 (3.06%; 70.3%). The corresponding measured numeric-domain medians (1129–1201 m/s) lie within the spread of five independently trained *θ* = 0.5 networks (1129–1225 m/s), so no effect of *θ* beyond training-realization variability is measurable. When *θ* is perturbed by ±0.1 at inference only, the measured median shifts by at most 1.1% on the network applied to the measured data. *θ* = 0.5 was therefore retained as a mid-range value.

### 3.2. Apparent-Velocity Extraction on the Measured Interval

The synthetically trained network was applied to the measured interval without retraining. In the numeric domain (2198 consecutive records), a median of 1200 m/s and an IQR of 72 m/s are obtained for the apparent quadrupole-mode velocity *V*_app_, and a median of 3.02 samples/channel is obtained for |*a*|. Because Δ*z*_eff_ was set empirically so that this median equals the adopted reference value of 1200 m/s, this level is reproduced by construction and provides no independent information on the absolute scale; with the nominal spacing of 0.100 m, the median would be 1654 m/s. In the image-inversion domain (2000 records; waveform pixel images, the same Δ*z*_eff_, no domain-specific retuning), the median is 1053 m/s with an IQR of 118 m/s, and a median of 3.44 samples/channel (about 69 µs) is obtained for |*a*|. The absolute level also depends on the training realization: numeric-domain medians of 1129–1225 m/s (about ±4%) are obtained with five independently trained networks with the same Δ*z*_eff_. The raw point-wise estimates scatter around these medians and are not free of record-to-record jumps. Because the records alternate between two acquisition states using period 2 (lag-1 waveform correlation ≈ 0.03; lag-2 ≈ 0.5–0.7), the same period-2 alternation is shown in the velocity output.

**Figure 7:**
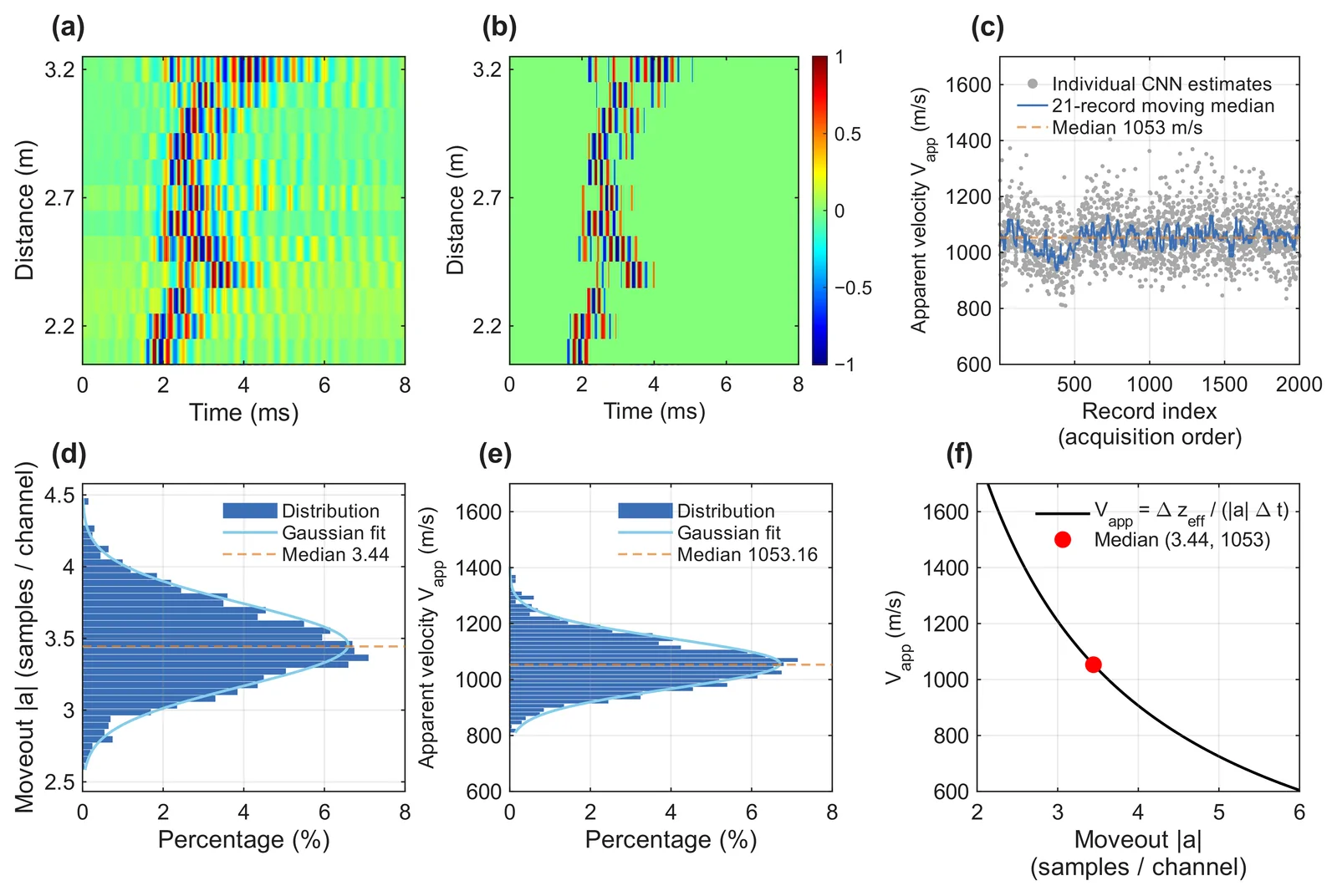
Apparent-velocity extraction from the measured interval. (**a**) Waveform pixel image. (**b**) Threshold-masked input (|*G*| ≥ 0.5). (**c**) Apparent-velocity log. (**d**) Inter-receiver moveout histogram. (**e**) Apparent-velocity histogram. (**f**) Hyperbolic velocity–moveout relation.

Figure 7 illustrates the image-inversion-domain extraction. Figure 7a shows a quadrupole waveform gather with a clear cross-receiver time shift of the dominant coherent wave packet, and Figure 7b shows the threshold-masked input; low-amplitude background is suppressed, while the principal moveout pattern is retained. Figure 7c shows the raw point-wise apparent velocity together with a centered 21-record moving median. This moving median is used only for display and is not part of the processing workflow; all statistics reported here and in the tables are computed from the raw point-wise values, and no post-processing is applied to any compared method. With this window, the medians change by at most 4 m/s, and the IQR falls to 36 and 45 m/s in the numeric and image-inversion domains, respectively. Because no depth field was stored, the vertical span of the window cannot be quantified precisely; responses of thin beds shorter than the window would be attenuated. Figure 7d shows the moveout distribution, centered at approximately 3.44 samples/channel, and Figure 7e shows the unimodal apparent-velocity distribution, with a median of 1053 m/s. Figure 7f shows the inverse moveout–velocity relation in Equation (1), including the median pair (3.44 samples/channel, 1053 m/s).

### 3.3. Cross-Domain Consistency

The measured-data comparisons are consistency and processing-sensitivity checks, not independent validation of absolute velocity. All variants are derived from the same measurements, and the numeric records are lossily re-encoded into rendered waveform plots in the image-inversion domain. Table 8 quantifies the agreement between the two domains. Before filtering, the medians are 1200.0 and 1053.2 m/s (difference +146.8 m/s [95% CI 141.7, 152.2], +13.9%), with a point-wise correlation of only 0.05. After the common 2.5–4.5 kHz band-pass, both medians are about 1168 m/s (difference +0.6 m/s [−6.8, 6.5]), the point-wise correlation rises to 0.36 [0.29, 0.42], and the median absolute difference falls from 147.8 m/s (14.1%) to 69.3 m/s (6.0%). The band-passed variants are processed by a separate network of the same architecture, retrained on synthetic gathers band-passed with the same filter.

**Figure 8:**
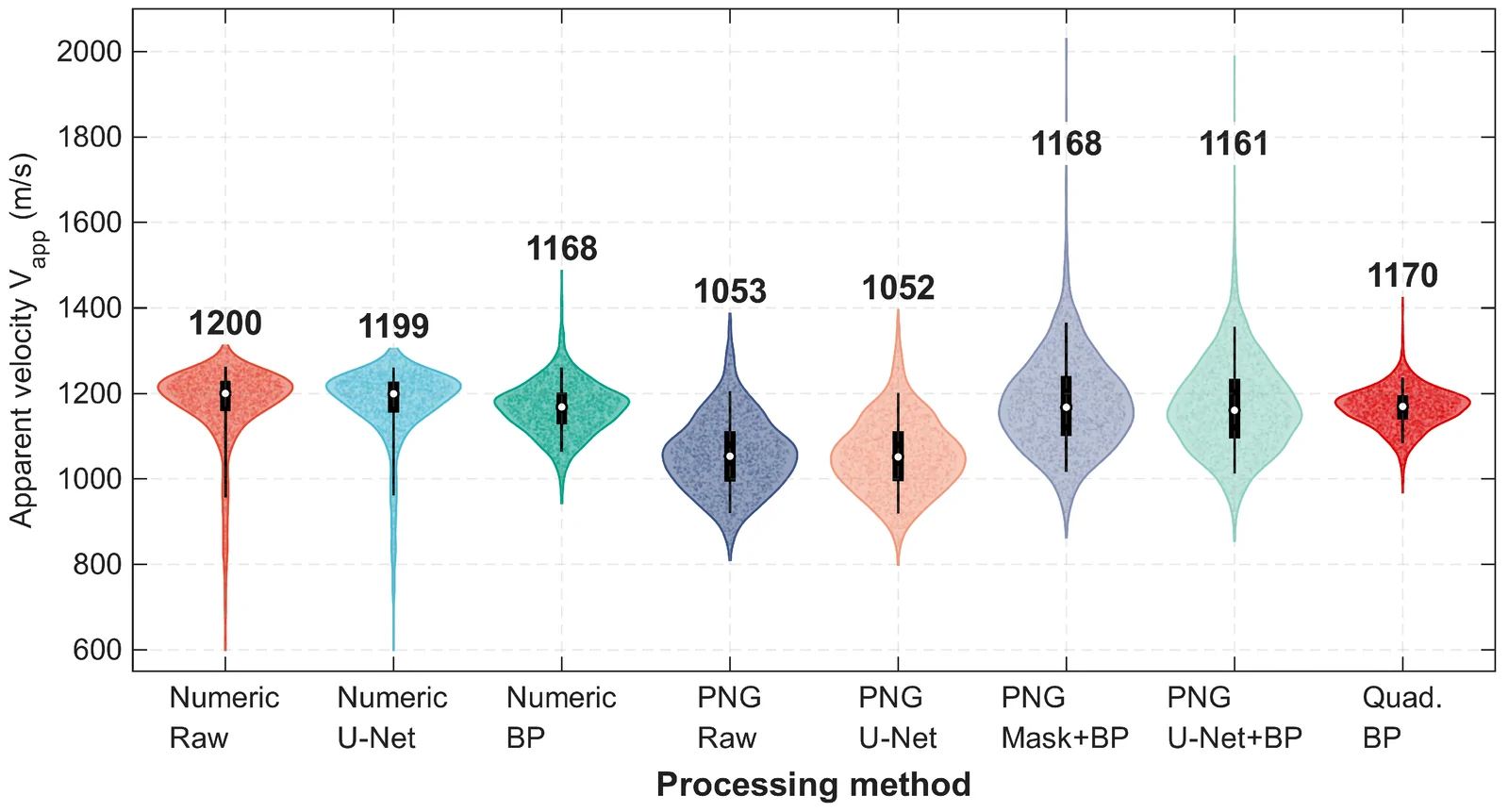
Apparent quadrupole-mode velocity distributions for eight processing variants (Numeric = numeric domain; PNG = image-inversion domain; Raw = threshold mask, no band-pass; U-Net = Attention U-Net mask; BP = 2.5–4.5 kHz band-pass; Quad. = quadrupole combination (S1 − S2 + S3 − S4)/4).

Because a depth field is stored in neither domain, records were paired by index with a one-record offset (image record i with numeric record i + 1), the offset at which waveform correlation is maximized (best match for 61% of records); the pairing is therefore statistical, and record identity is not proven. Correlations of the smoothed trends (21-record moving median) are 0.27 before and 0.38 after band-pass, and for windows of 51 records or longer, the trend agreement is higher before band-pass. The median offset is thus removed, and band-pass filtering, together with the band-pass-specific network, improves point-wise agreement, but long-wavelength agreement does not improve; because the network also changes between the two rows of Table 8, the effects of filtering and retraining are not separated. Figure 8 shows the apparent-velocity distributions for eight processing variants covering domain representation, band-pass filtering, mask type, and quadrupole combination: domain representation and frequency band cause the greatest shift in the distributions, whereas mask type and quadrupole combination change the median by less than 1%.

**Figure 9:**
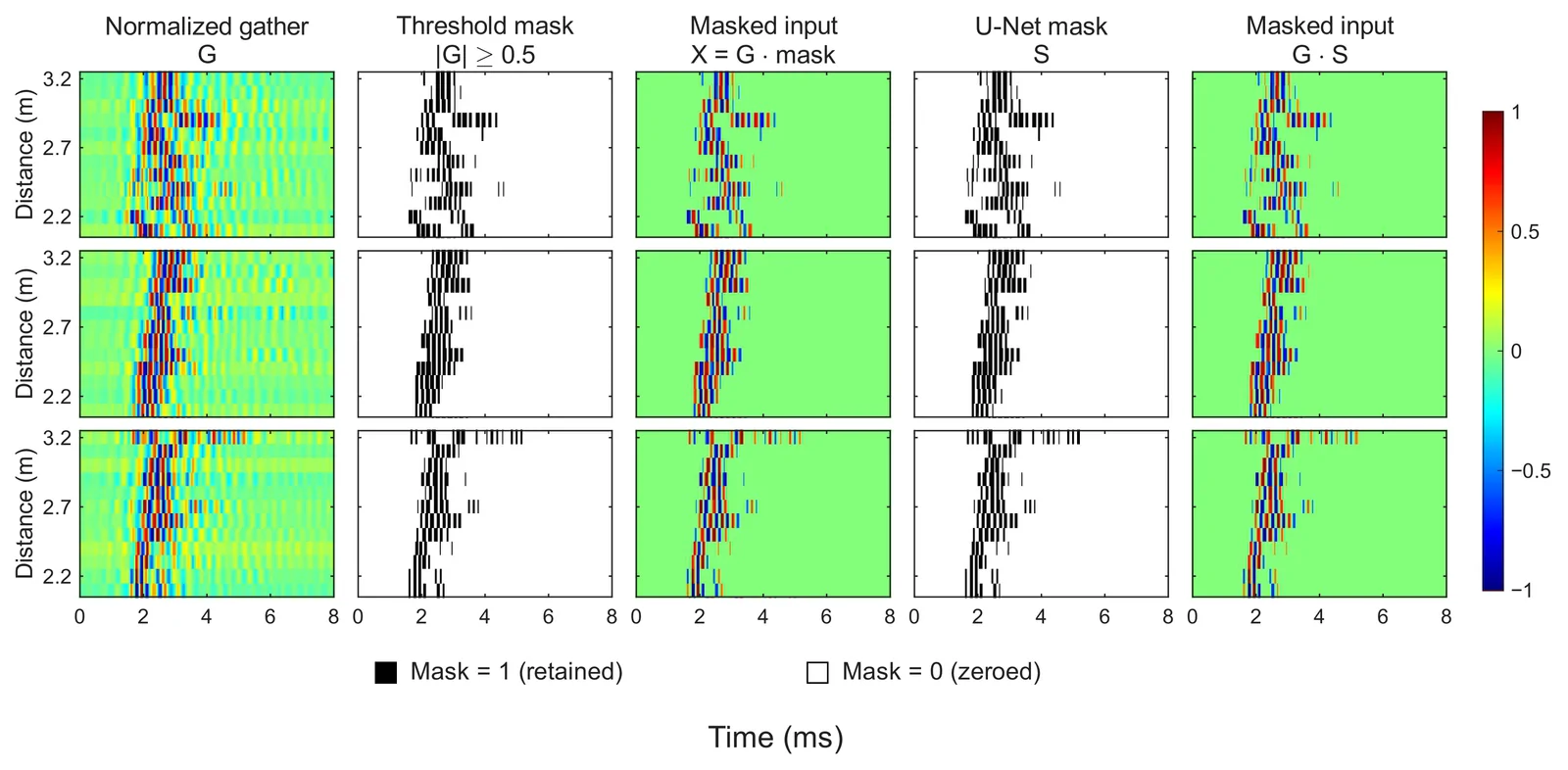
Threshold mask versus Attention U-Net segmentation mask.

In Figure 9, the threshold mask is compared with an Attention U-Net segmentation mask (about 0.49 M parameters). The U-Net reproduces the threshold rule, as shown by the per-image IoU of about 0.97 and the median differences (1199 versus 1200 m/s; 1052 versus 1053 m/s; band-passed 1161 versus 1168 m/s, i.e., 0.6%). This is expected because its training labels were generated by the same rule (|*G*| ≥ 0.5), so the comparison is not an independent check; it indicates only that nothing is added by a U-Net trained to imitate the threshold rule. We did not test whether a front-end trained with independent labels would change the result, but the fixed threshold keeps the front-end free of trainable weights. A band-passed median of 1170 m/s is obtained with the quadrupole combination (S_1_ − S_2_ + S_3_ − S_4_)/4 of the four quadrupole components versus 1168 m/s for the recorded quadrupole trace; individual components were not compared. The self-computed STC (configuration in Table 2), applied to numeric records filtered to 2.5–4.5 kHz with a Kalman resonator-bank filter rather than the Butterworth band-pass used for the CNN variants, yields a median of 1051 m/s with a wide IQR of 435 m/s.

**Figure 10:**
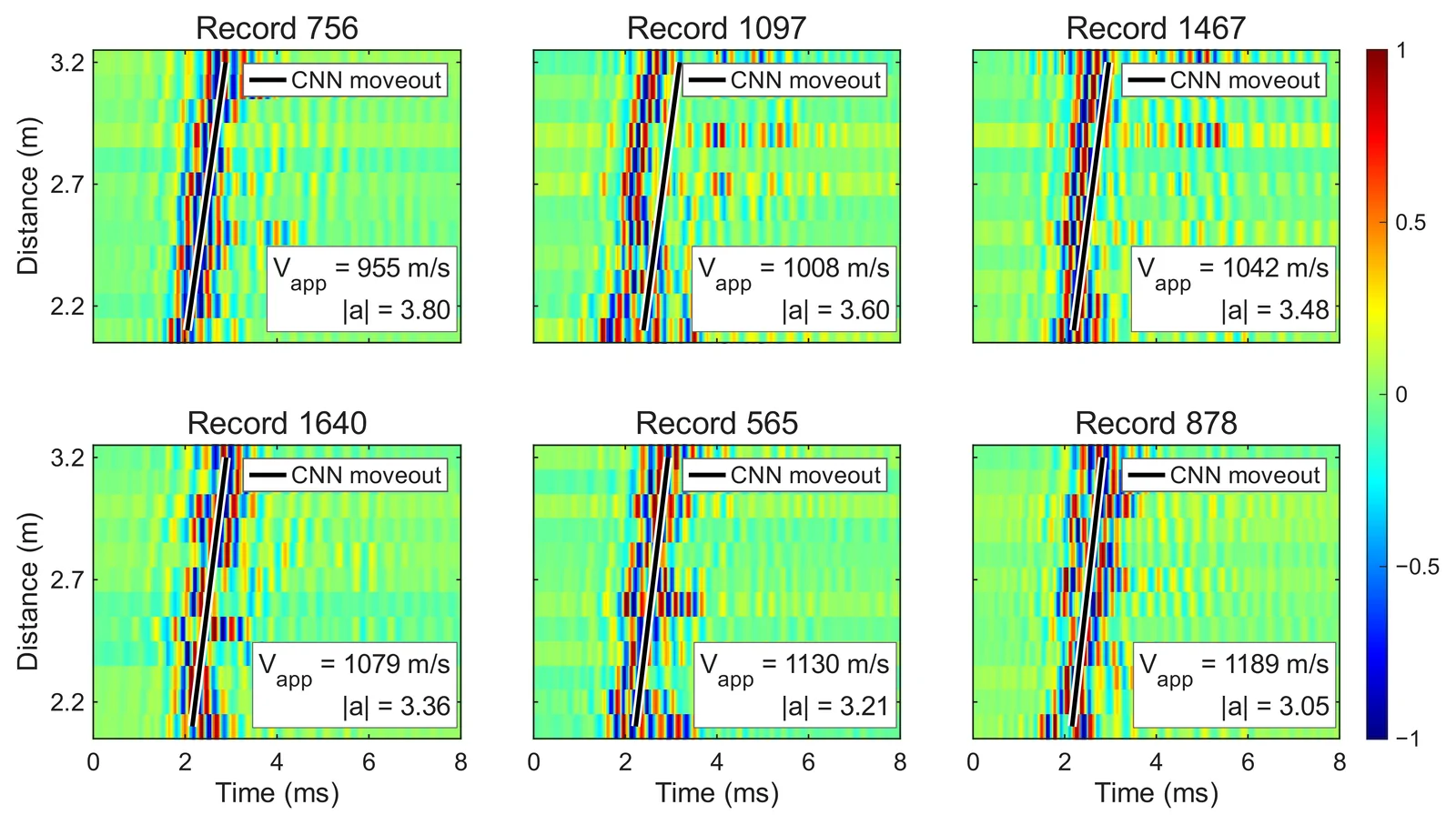
CNN regression-based extraction on example image-inversion-domain gathers.

Figure 10 shows examples on image-inversion-domain gathers: the high-amplitude coherent event is followed across traces by the line implied by the CNN-regressed moveout, suggesting that for these selected examples, the estimate follows the cross-trace slope of the dominant wave packet; this was not quantified over the interval. Against the measured STC log on the 1032 paired records, the image-domain median is 54 m/s lower (−4.9%), but the point-wise correlation is only 0.09, so agreement is limited to central tendency. Because Δ*z*_eff_ was set empirically, the absolute velocity scale cannot be corroborated by this agreement.

### 3.4. Comparison Tests Against the Measured STC

With random splitting (826 training and 206 held-out records), the curve follows the central trend and compresses the global spread, as expected for a variant supervised by the measured STC curve. On the 206 held-out records, which were also used for model selection, the ratio of the median prediction to the median measured STC is 1.008, the median relative error is 7.22%, the fractions within 5%/10% are 34%/65%, and *r* = 0.53, which is better than the constant baseline set to the training-label median (9.03%). Over the full interval (including the 826 training records), the global spread of its velocities is smaller than that of the measured STC: the standard-deviation ratio is 0.74, and the IQR ratio is 0.85; *p* = 1.9 × 10^−14^ is obtained with a Brown–Forsythe test, but the two series are treated as independent samples, and their pairing and serial correlation are ignored, so this *p*-value is indicative only.

**Figure 11:**
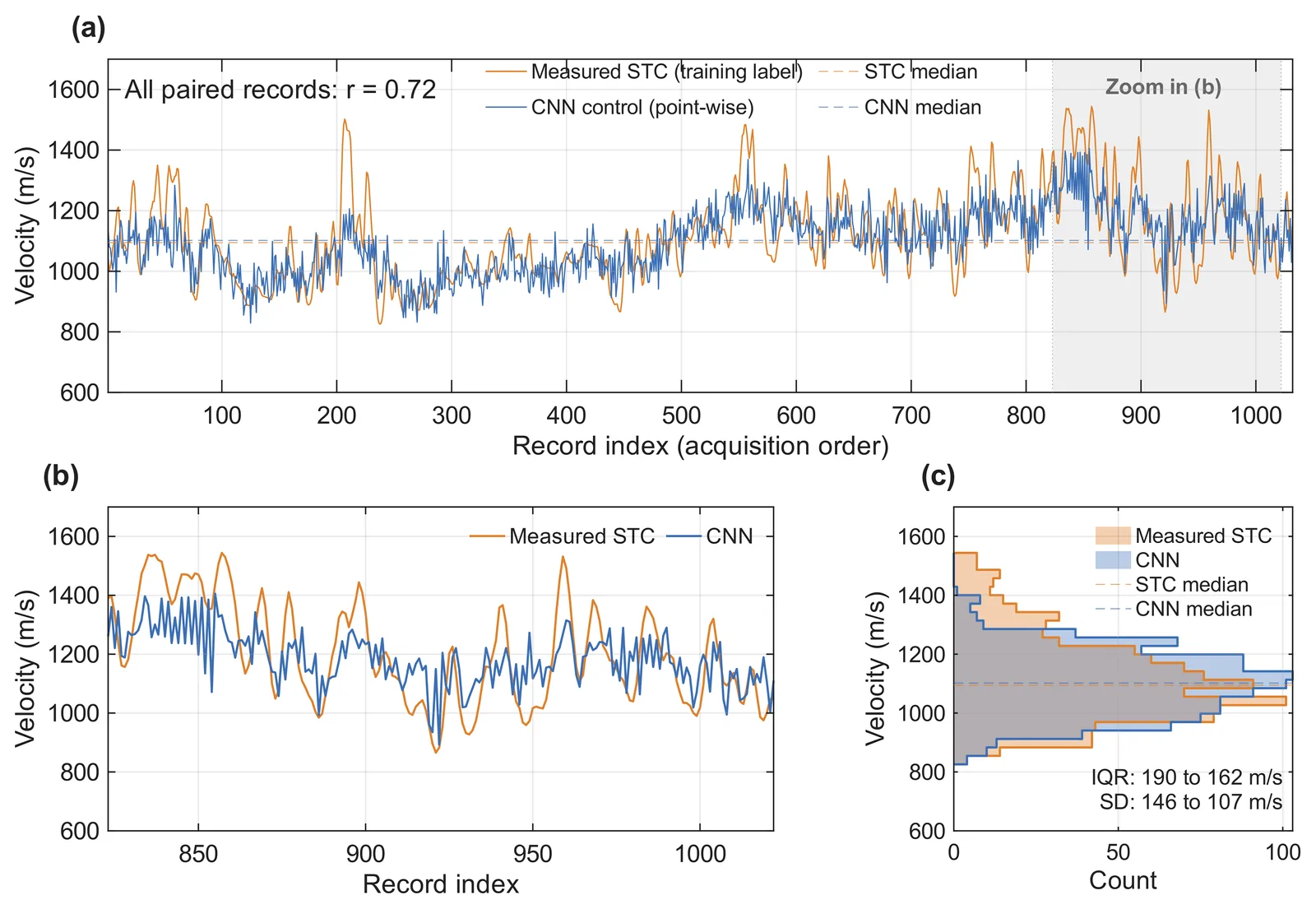
Measured-STC-supervised comparison variant versus measured STC curve. (**a**) Velocity curves. (**b**) Most informative interval. (**c**) Velocity distributions.

The variant is compared with the measured STC curve in Figure 11, i.e., (a) the two curves over the full interval, with the central trend of the measured STC followed by the variant; (b) a zoom-in on the most informative interval, where the variant passes through the large label fluctuations without tracking them point by point; (c) the velocity distributions, with close medians but a narrower spread for the variant. This behavior is consistent with MSE training, where the network approximates an MSE-optimal regression mapping over the training distribution rather than reproducing the labels point by point. Using depth-blocked five-fold cross-validation, which removes near-neighbor leakage, we show that random splitting’s apparent learnability is supported mainly by such leakage. Under blocking, the pooled out-of-fold correlation drops from 0.53 to 0.20 (mean of per-fold r: 0.006, range: −0.15 to 0.17), and the median relative error rises to 11.93%, worse than the blocked constant baseline (9.42%). In Table 9, mean per-fold blocked correlations of 0.04–0.16 are reached by the five classical regression models, trained with the same inputs, labels, and splits, and all are higher than the 0.006 of the CNN. Blocked median errors below the constant baseline (9.42%) are obtained with three of them (random forest, ExtraTrees, HistGBM: 8.80–9.07%), whereas the CNN (11.93%) results are worse than this baseline.

Table 9 summarizes the comparison of the variant and the classical regression models under both protocols. Switching from random to depth-blocked splitting degrades all models, and the gap between the two protocols quantifies the contribution of leakage to the apparent accuracy. The variant’s residuals also show a systematic, velocity-dependent bias. Over the full interval, the signed median residual is approximately −14 m/s (about −1.3%); however, in terciles of the measured STC velocity, the signed bias is +1.76% for the slow tercile (826–1036 m/s), +0.93% for the intermediate tercile (1036–1159 m/s), and −5.74% for the fast tercile (1159–1544 m/s), with per-tercile median relative errors of 5.27%, 4.84%, and 7.08%, respectively. This pattern is consistent with shrinkage toward the conditional mean under MSE regression, but it is also produced mechanically by stratifying on the noisy STC labels themselves (regression to the mean), even for an unbiased predictor of the true velocity; a network bias is therefore not isolated by the tercile biases.

**Figure 12:**
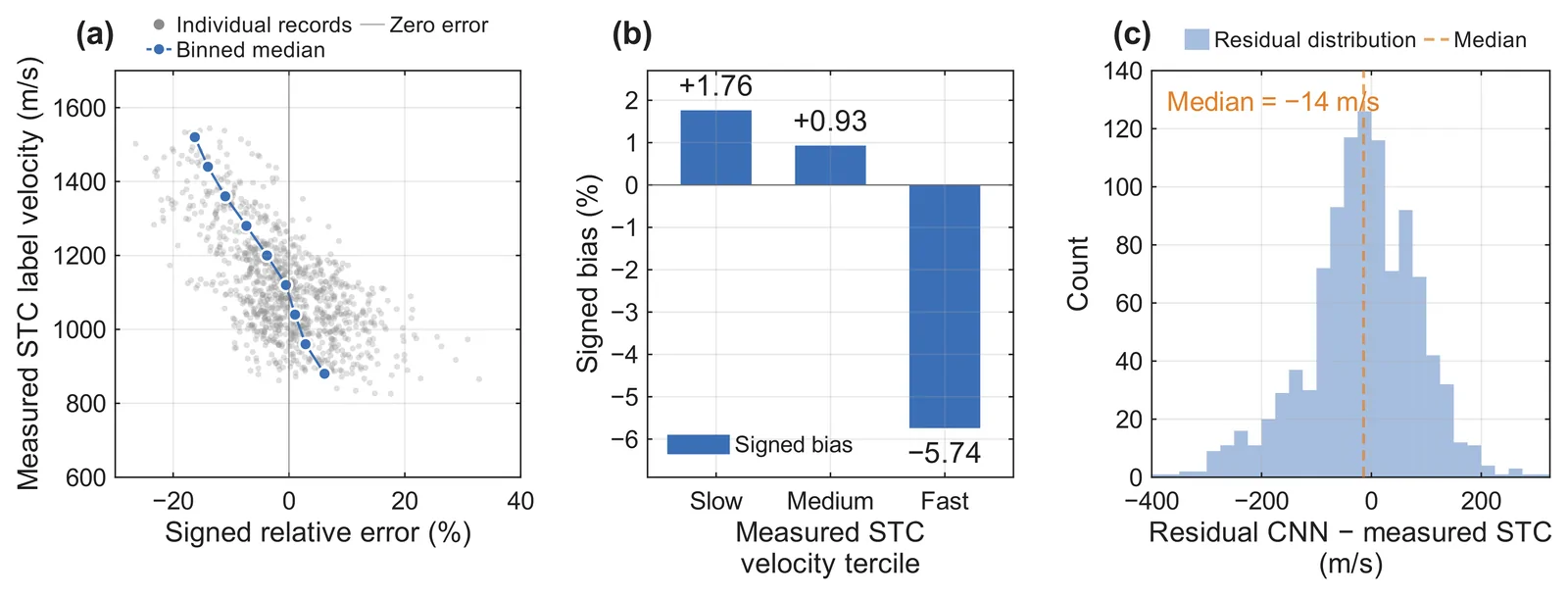
Error structure of the comparison variant. (**a**) Binned median signed relative error. (**b**) Signed bias by velocity tercile. (**c**) Residual (CNN-measured STC) histogram.

Structural bias is not ruled out by approximate unbiasedness overall and must be examined by velocity stratification. The residual structure of the comparison variant is dissected in Figure 12: (a) the binned median of the signed relative error is shown as a function of measured STC velocity, with the error varying monotonically with velocity; (b) the results are summarized by slow/intermediate/fast terciles, with a bias of +1.76% for the slow tercile, +0.93% for the intermediate tercile, and as much as −5.74% for the fast tercile—a shrinkage pattern of overestimation at the slow end and underestimation at the fast end; (c) the residual histogram is shown, with a full-interval signed median residual of approximately −14 m/s (about −1.3%), approximately unbiased overall. For the population MSE-optimal mapping, it is implied by Equation (10) that the label variance is not exceeded by the prediction variance, and the weaker the explanatory power of the input for the labels, the stronger the shrinkage toward the conditional mean; this mapping is only approximated by a finite network trained on finite data, but the stratified bias is consistent with this global shrinkage projected onto the velocity axis. This implies that when downstream interpretation depends on absolute velocity levels, this velocity-dependent systematic bias must be accounted for.

**Figure 13:**
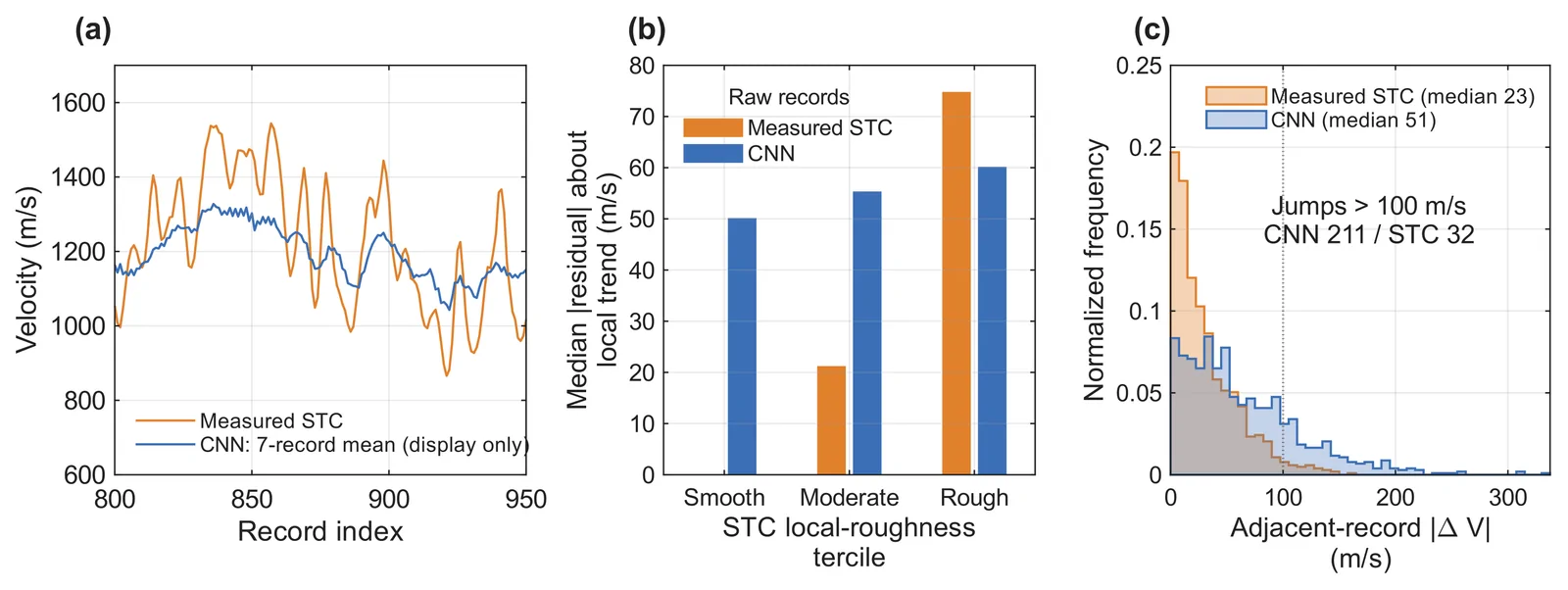
Point-wise behavior of the comparison variant. (**a**) Measured STC and CNN velocity curves. (**b**) Median residuals regarding the local trend. (**c**) Adjacent-record |ΔV| distributions (CNN and measured STC).

Figure 13 examines the point-wise behavior of the measured-STC-supervised CNN. As shown in Figure 13a, the CNN generally does not follow the large excursions in the measured STC labels. At the 153 records with the largest STC deviations from the local trend, the median absolute residual is reduced from 112 to 66 m/s by the CNN, which is closer to the local trend at 76% of these points (Figure 13b); because these records were selected for large STC deviations, and training records are included, such a reduction would be shown by any smoother predictor, so this result is descriptive only. Between adjacent records (Figure 13c), the median velocity jump is 51 m/s for the CNN and 23 m/s for the measured STC, with 211 and 32 jumps exceeding 100 m/s, respectively. At lag 2, i.e., between records of the same acquisition state, the corresponding values are 47 and 42 m/s, with 159 and 196 jumps exceeding 100 m/s. The locally noisier appearance of the CNN at lag 1 is therefore largely due to the period-2 alternation of the records. Overall, the CNN yields a tighter global distribution, and it does not follow the large STC excursions, consistent with regression toward the conditional mean rather than pointwise denoising.

### 3.5. Computational Cost

The computational cost of the 1032 image-domain gathers is summarized in Table 10 (warm-up followed by ten repetitions; mean ± SD). One deterministic forward pass is performed per gather by the CNN (68,065 parameters; 266 KB model file). With a batch size of 256 on an GPU, the total time was 29.5 ± 4.4 ms (0.029 ms per gather), including host–device transfer but excluding masking; with a batch size of 1, the latency was 0.73 ms per gather. For batched CNN inference on the same, 183.5 ± 4.9 ms was required; for STC with 16 workers, searching 1000–3500 m/s at 0.100 m (≡725–2538 m/s at 0.0725 m, a wider grid than that of Table 6), 632.7 ± 8.3 ms was required with 25 m/s steps and 2977 ± 41 ms with 5 m/s steps. Different hardware and batched throughput are compared by the GPU-CNN to a 25 m/s STC ratio of about 21×, which is not an algorithmic speedup; at a batch size of 1, the GPU latency (0.73 ms per gather) is comparable to the per-gather throughput of a 16-worker STC (0.61 ms); on the same CPU, the CNN is 3.4× faster than the 25 m/s STC and 16× faster than the 5 m/s STC. The repeated velocity–time grid search of STC is replaced by the fixed-cost forward pass, and the approximately 0.49 M-parameter Attention U-Net front-end is avoided through the threshold mask. Embedded hardware, power consumption, activation memory, and end-to-end preprocessing were not evaluated; therefore, only potential suitability for downhole or near-sensor edge implementation is indicated by the results, and hardware-in-the-loop testing is required.

## 4. Discussion

### 4.1. Interpretation

In this work, we replace the scan-and-pick step of STC with a direct regression of the inter-receiver moveout *a*, learned from kinematic synthetic gathers. Under a linear arrival-time model, *a* is independent of absolute arrival time and amplitude scale, and a single parameter is introduced by conversion to velocity, i.e., the effective receiver spacing Δ*z*_eff_. Under MSE training (Equation (7)), the network approximates an MSE-optimal regression mapping over the training distribution, without guarantees outside it.

In distribution (Table 6), the CNN produced fewer gross errors than did the STC (0.50% versus 3.88%; paired difference −3.38 percentage points [−4.88, −2.00]), but with a slightly larger median error (2.11% versus 1.57%). Because the STC gross-error rate was not reduced by a finer grid, these errors are wrong-peak selections. STC’s explicit peak-switching mechanism is therefore removed, and continuous regression reduces an important source of abrupt outliers. However, large errors still occur under severe interference or distribution shift: outside the training range (*A*_s_/*A*_t_ ≥ 0.75 or SNR ≤ 5 dB; Table 7), CNN errors grew rapidly, reaching 20.88% and 25.88% gross errors at *A*_s_/*A*_t_ = 1.00 and SNR = −5 dB, whereas STC remained robust to additive noise. STC thus remains a useful cross-check.

The measured-data checks establish consistency rather than independent validation. The image-inversion domain is a lossy re-encoding of the same measurements; after a common 2.5–4.5 kHz band-pass, the two domain medians agree (1168.2 versus 1167.6 m/s), but pointwise agreement remains moderate (*r* = 0.36; Table 8). The Attention U-Net was trained with labels from the same threshold rule, so its IoU of about 0.97 reflects reproduction, not confirmation, of the rule. Thus, only the central tendency of the moveout is reproducible across representations of the same data, and only after band-pass filtering with a band-pass-specific network; point-wise agreement is weak (*r* = 0.05 before and 0.36 after band-pass), and absolute velocity is not validated.

On the scale side, the nominal receiver spacing is 0.100 m, whereas velocities were converted with Δ*z*_eff_ = 0.0725 m. This value was set empirically, scaling the numeric-domain median obtained with 0.100 m (1654 m/s) to a reference value of 1200 m/s; it was neither derived theoretically nor calibrated on an independent interval, and it was fixed after the measured STC log had been examined. The 1200 m/s median is therefore reproduced by construction, and no comparison with the measured STC can corroborate the absolute scale. Velocity changes by 1.38% per 1 mm change in spacing, and the absolute level varies by about ±4% across independently trained networks.

Compared with synthetic-to-measured transfer [19,20,21] and direct slowness regression from the LWD waveforms [22], the difference is the supervision target, i.e., moveout rather than velocity, with the absolute scale isolated in one parameter. Unlike physics-informed dispersive processing [23,24], dispersive STC [15], and high-resolution dispersion inversion [16], this method does not model dispersion. These dispersion-aware methods require tool and borehole parameters that were unavailable and thus, were not implemented; multi-scale STC [9] was also not implemented. The comparison in Table 6, therefore, limits us to non-dispersive estimators on kinematic synthetic gathers, and we do not test the CNN’s lower gross-error rate against multi-scale or dispersive STC.

The measured-STC-supervised control (Table 9) behaves as expected for distillation from possibly noisy pseudo-labels [33,34,35]. Its held-out correlation under random splitting (*r* = 0.53) fell to 0.20 (pooled out-of-fold; mean per-fold *r* = 0.006) under depth-blocked cross-validation, and its median error (11.93%) was higher than that of the blocked constant baseline (9.42%). Performance is thus overestimated by random splits through near-neighbor leakage. Its narrower global spread is consistent with regression shrinkage (Equation (10); [44]), and the period-2 alternation of acquisition states is largely reflected in its larger adjacent-record jumps (at lag 2, 47 versus 42 m/s for STC).

The 21-record moving median in Figure 7c is for display only; it is not part of the processing workflow, and all reported statistics are raw, point-wise values for all methods. Because thin-bed or rapidly varying responses shorter than the window would be attenuated by such smoothing, its use is not recommended for delivered logs.

### 4.2. Limitations

First, the training data are kinematic synthetic gathers (Table 4). We omit frequency-dependent dispersion, collar waves, attenuation, tool eccentricity, borehole geometry, formation elastic parameters, and modal interference. No domain adaptation [21] was applied; the synthetic-to-field gap is addressed only by domain randomization [42] and is not quantified. Second, we assume non-dispersive propagation when converting a single moveout to velocity, whereas the quadrupole mode is dispersive and approaches the formation shear-wave velocity only in the low-frequency limit [15,16]. The output is therefore an apparent quadrupole-mode velocity, and its relation to the formation shear-wave velocity is not established: velocities are lowered by the empirical Δ*z*_eff_ relative to the nominal 0.100 m, opposite to the direction of a dispersion correction, so it is an unexplained scale factor rather than a physical correction.

Third, a single well interval without independent ground truth is covered by the field evaluation, i.e., wireline dipole, core, or laboratory ultrasonic velocities were unavailable; the measured STC itself is not ground truth. Fourth, the three measured data sets differ in size (2198 numeric records, 2000 archived plots, and 1032 records with a commercial STC value), and no record-to-depth correspondence was stored; numeric and image records are paired statistically, and vertical resolution cannot be stated in meters. Finally, the records alternate between two acquisition states with period 2; this alternation also appears in the velocity output.

### 4.3. Implications and Future Work

Potential, rather than demonstrated, suitability for edge deployment is indicated by the 266 KB model and the single forward pass. Four routes will be pursued in future work: (1) Δ*z*_eff_ will be calibrated on intervals with independent ground truth from cores or wireline dipole logging; (2) the kinematic generator will be replaced with full-waveform quadrupole modeling, domain adaptation will be added, and transfer across wells will be tested under depth-blocked validation; (3) a dispersion-aware moveout-to-velocity conversion will be introduced and compared with dispersive and multi-scale STC [9,15,16] and physics-informed routes [23,24]; (4) INT8 quantization and hardware-in-the-loop deployment [45,46] will be evaluated with end-to-end timing and power.

## 5. Conclusions

A lightweight CNN trained only on kinematic synthetic gathers with known moveout was proposed for regression of the inter-receiver moveout of quadrupole acoustic LWD array gathers. The estimated moveout is converted to an apparent quadrupole-mode velocity via Equation (1) using the effective receiver spacing in Table 2. The main findings are as follows:

1. On synthetic data, a median relative velocity error of 2.51% ± 0.16% was obtained by five-fold cross-validation, and an error of 2.11% with 0.50% gross errors was obtained on the held-out test. On the same gathers, the CNN achieved fewer gross errors (0.50% versus 3.88%) but a slightly higher median error (2.11% versus 1.57%), and it outperformed cross-correlation, GCC-PHAT, and robust fitting. Stress tests showed rapid degradation outside the training distribution (secondary-to-target amplitude ratio ≥ 0.75, SNR ≤ 5 dB), and STC was more robust to additive noise.
2. On a measured quadrupole acoustic LWD interval of a physical experimental well in a slow formation, an apparent-velocity log with a numeric-domain median of 1200 m/s and an IQR of 72 m/s was produced by this method. This absolute level follows from the empirically set effective spacing (0.0725 m versus a nominal 0.100 m; 1 mm corresponds to 1.38% in velocity) and varies by about ±4% across training realizations, so it is not independently validated. After a common 2.5–4.5 kHz band-pass, the numeric and image-inversion domains agreed in median (difference 0.6 m/s) but were only in moderately pointwise agreement (*r* = 0.36); both are derived from the same measurements. A control supervised by the measured STC reached r = 0.53 on held-out records under random splitting, but only 0.20 (mean per-fold 0.006) under depth-blocked cross-validation, showing limited generalization of STC-derived labels across contiguous record blocks.
3. Inference consists of a single deterministic forward pass with a 266 KB model (68,065 parameters). In the tested desktop configurations, GPU inference was about 21× faster than a 16-worker CPU STC with a 25 m/s grid, and 3.4× faster when both were run on the same CPU. Embedded hardware, power consumption, and end-to-end preprocessing were not evaluated, so suitability for edge deployment remains potential.

In summary, we obtain a fast apparent-velocity extractor through synthetically supervised moveout regression; it requires no measured labels, and it suppresses wrong-peak gross errors within its training distribution. To convert its output to formation shear-wave velocity requires independent calibration of the effective spacing against core or wireline dipole data, dispersion-aware conversion, physically simulated training data, and validation across multiple wells.

## Figures and Tables

**Table 1:** Representative acoustic shear-wave processing methods.

Method	Approach	Output	Main Feature
Conventional STC [8,9]	Coherence scanning	Slowness	Susceptible to peak switching
Dispersive processing [15,16]	Dispersion analysis/inversion	Shear slowness	Dispersion is explicitly considered
Deep-learning inversion [22]	Data-driven inversion	Formation slowness	Fast LWD acoustic inversion
Physics-informed DL [23,24]	Physics-informed learning	Dispersion characteristics	Dispersion physics is incorporated
Proposed CNN	Synthetic moveout regression	Moveout/apparent velocity	Lightweight; few parameters

**Table 2:** Acquisition and processing constants.

Quantity	Symbol	Value
Number of receiver channels	*N*_ch_	12
Nominal receiver spacing	Δ*z*_nom_	0.100 m
Effective spacing for velocity conversion	Δ*z*_eff_	0.0725 m
Time sampling interval	Δ*t*	20 µs
Time samples used per trace	*N*_t_	400 (of 1024)
Threshold-mask level	*θ*	0.5 (normalized amplitude)
Band-pass for processing variants	—	2.5–4.5 kHz
Numeric-domain records	—	2198
Image-inversion records	—	2000
Records covered by the measured STC log	—	1032 (random split 826/206)
Synthetic training/validation/test gathers	—	4000/800/800
Calculated STC speed range	—	1000–3500 m/s
STC reference search: time window	—	45 samples (0.9 ms)

**Table 3:** Regression CNN layers.

Layer	Kernel/Units	Output Size
Input	—	12 × 400 × 1
Conv + BN + ReLU	3 × 7, 16	12 × 400 × 16
MaxPool	2 × 2, stride 2	6 × 200 × 16
Conv + BN + ReLU	3 × 5, 32	6 × 200 × 32
MaxPool	2 × 2, stride 2	3 × 100 × 32
Conv + BN + ReLU	3 × 3, 64	3 × 100 × 64
MaxPool	1 × 2, stride [1 2]	3 × 50 × 64
Conv + BN + ReLU	3 × 3, 64	3 × 50 × 64
Global average pooling	—	64
Fully connected + ReLU	64	64
Dropout	*p* = 0.2	64
Fully connected (linear)	1	1 ($\left|\hat{a}\right|$, samples/channel)

**Table 4:** Kinematic synthetic gather-generator parameters.

Parameter	Symbol	Value/Range
Receiver channels	*N*_ch_	12
Samples per trace	*N*_t_	400
Sampling interval	Δ*t*	20 µs
Spacing for velocity–moveout conversion	Δ*z*_eff_	0.0725 m
Target velocity	*V*	U(760, 1520) m/s
Target moveout	|*a*|	2.38–4.77 samples/channel, single polarity
Earliest-channel onset	*t*_0_	40–110 samples (0.8–2.2 ms)
Wavelet	—	Gabor, 3–6 cycles, random phase
Wavelet peak frequency	*f*_p_	U(2.3, 3.0) kHz
Per-channel gain	—	1 + 0.06·N(0, 1)
Amplitude decay across array	—	Linear, 0–25%
Secondary events per gather	—	1 or 2
Secondary-event velocity	—	U(760, 1520) m/s
Secondary-event onset (channel 1)	—	Earliest target arrival + 25–135 samples
Secondary/target amplitude ratio	*A*_s_/*A*_t_	U(0.20, 0.55), ±8% per-channel jitter
Secondary-event center frequency	—	*f*_p_·U(0.8, 1.2)
Coda (probability 0.5)	—	Gaussian-enveloped noise, 0.08 rms
Noise	—	Band-limited Gaussian, 1.25–15 kHz
Signal-to-noise ratio (whole-gather RMS)	SNR	U(10, 22) dB
Normalization	—	Per-gather max-abs; no DC removal
Training/validation/test gathers.	—	4000/800/800

**Table 5:** CNN metrics on the held-out synthetic test set.

Metric	Value
Coefficient of determination, *R*^2^	0.947
Pearson correlation coefficient, *r*	0.974
Spearman rank correlation, *ρ*	0.975
Concordance correlation coefficient, CCC	0.973
Root-mean-square error, RMSE (m/s)	51
Mean absolute error, MAE (m/s)	35
Mean absolute percentage error, MAPE (%)	3.02
Median relative error (%)	2.11
Signed median bias (%)	+0.63
Fraction within 5%/10%/15% (%)	83.0/96.5/98.9
Gross-error (>20%) fraction (%)	0.50
P90/P95 |relative error| (%)	6.8/9.0

**Table 6:** Moveout estimators on the 800 held-out synthetic gathers.

Method	MAE (m/s)	Median Error (%) [95% CI]	Within 5% (%)	Within 10% (%)	Gross Error > 20% (%)	*r*
Cross-correlation	78.0	5.74 [5.38, 6.18]	43.5	77.4	0.63	0.894
GCC-PHAT (1.5–4.5 kHz)	101.9	6.86 [6.44, 7.43]	37.9	66.1	9.38	0.802
Robust fit (envelope peak + Theil–Sen)	205.7	7.65 [6.99, 8.41]	34.0	58.8	16.88	0.298
STC, 25 m/s grid	40.6	1.57 [1.45, 1.68]	86.6	93.5	3.88	0.915
STC, 5 m/s grid	37.9	1.30 [1.21, 1.39]	88.4	93.9	3.75	0.915
Proposed CNN	34.8	2.11 [1.89, 2.29]	83.0	96.5	0.50	0.974

Note: Raw, unmasked gathers are used by classical methods.

**Table 7:** Stress tests on the 800 held-out synthetic seeds: secondary-event amplitude ratio and SNR.

Condition	CNN Median Error (%)	STC Median Error (%)	CNN Gross Error (%)	STC Gross Error (%)
**Secondary amplitude ratio**				
*A*_s_/*A*_t_ = 0	1.58	1.12	0.0	0.0
*A*_s_/*A*_t_ = 0.25	1.69	1.40	0.0	0.75
*A*_s_/*A*_t_ = 0.50	2.74	1.78	1.0	6.63
*A*_s_/*A*_t_ = 0.75	6.59	2.56	8.5	15.88
*A*_s_/*A*_t_ = 1.00	10.15	4.66	20.88	23.5
**SNR**				
SNR = 22 dB	2.18	1.66	0.63	6.63
SNR = 16 dB	2.07	1.57	0.63	3.75
SNR = 10 dB	2.32	1.48	0.63	1.50
SNR = 5 dB	3.45	1.48	0.50	0.50
SNR = 0 dB	7.43	1.65	5.38	0.13
SNR = −5 dB	12.13	2.06	25.88	0.13

Note: CNN = identical-protocol retrain; STC grid 25 m/s; gross error > 20%.

**Table 8:** Agreement between numeric-domain and image-inversion-domain apparent velocities.

Comparison	Numeric Median (m/s)	Image Median (m/s)	Median Difference (m/s) [95% CI]	Relative Difference (%)	Pointwise r [95% CI]	MAE (m/s)	Median |Δ| (m/s)
Before band-pass	1200.0	1053.2	+146.8 [141.7, 152.2]	+13.9	0.05 [−0.01, 0.12]	151.0	147.8 (14.1%)
After 2.5–4.5 kHz band-pass	1168.2	1167.6	+0.6 [−6.8, 6.5]	+0.05	0.36 [0.29, 0.42]	83.7	69.3 (6.0%)

Note: All records are used for medians and median differences (2198 numeric, 2000 image; 95% CIs from 5000 i.i.d. bootstrap resamples); the 2000 index-paired records (image i ↔ numeric i + 1) are used for *r* (moving-block bootstrap CI, block length 50), MAE and median |Δ|.

**Table 9:** Measured-STC-supervised comparison variant versus classical regression models.

Model	Random MRE	Random *r*	Blocked MRE	Blocked *r*
CNN threshold-mask (comparison variant)	7.22%	0.53	11.93%	0.20
ExtraTrees (300)	5.57%	0.66	8.94%	0.13
Random forest RF(300)	6.79%	0.62	8.80%	0.14
HistGBM	6.56%	0.62	9.07%	0.16
SVR(RBF)	6.78%	0.57	9.66%	0.11
Ridge regression	6.82%	0.56	10.24%	0.04
Constant baseline	9.03%	—	9.42%	—

Note: Blocked values are pooled out-of-fold, for the CNN and constant baseline (per-fold means: CNN *r* = 0.006, MRE = 12.17%; baseline MRE = 9.48%), with per-fold means for the classical models.

**Table 10:** Computational cost of the 1032 image-domain gathers (mean ± SD of ten repetitions).

Method	Device	Mode	Total for 1032 Gathers (ms)	Per Gather (ms)	Model Size
CNN	GPU	Batch 256	29.5 ± 4.4	0.029	266 KB
CNN	GPU	Batch 1 (latency)	751 ± 143	0.73	266 KB
CNN	CPU (16 threads)	Batch 256	183.5 ± 4.9	0.18	266 KB
STC (25 m/s step)	CPU (16 workers)	Parallel grid search	632.7 ± 8.3	0.61	—
STC (5 m/s step)	CPU (16 workers)	Parallel grid search	2977 ± 41	2.88	—

Note: Timed after one warm-up run; host–device transfer is included in GPU times, and masking is excluded.

## Data Availability

The measured dataset supporting this study was acquired in a quadrupole acoustic LWD experiment performed on the physical experimental well at the National Key Laboratory of Deep Oil and Gas, China University of Petroleum (East China); together with the measured STC log, it is subject to confidentiality restrictions and cannot be deposited publicly, but it can be obtained from the corresponding author upon reasonable request and subject to agreement. The kinematic synthetic gathers can be reproduced from the generator settings in Table 4, the acquisition and processing constants in Table 2, and the network architecture in Table 3; random seeds and data partitions are also provided. The code, synthetic generator, trained weights (the identical-protocol retrain of the Table 5 network, the network applied to the measured data, and the band-pass network), and synthetic data will be made available to the editor and reviewers on request during review and will be deposited in a public repository upon publication.
